# PKCμ promotes keratinocyte cell migration through Cx43 phosphorylation-mediated suppression of intercellular communication

**DOI:** 10.1016/j.isci.2024.109033

**Published:** 2024-01-29

**Authors:** Renju Pun, Ann M. Cavanaugh, Emily Aldrich, Olivia Tran, Justin C. Rudd, Laura A. Hansen, Brian J. North

**Affiliations:** 1 Biomedical Sciences Department, School of Medicine, Creighton University, Omaha, NE 68178, USA; 2 Department of Biology, College of Arts and Sciences, Creighton University, Omaha, NE 68178, USA

**Keywords:** Biological sciences, Molecular biology, Molecular interaction, Cell biology

## Abstract

Downregulation of intercellular communication through suppression of gap junctional conductance is necessary during wound healing. Connexin 43 (Cx43), a prominent gap junction protein in skin, is downregulated following wounding to restrict communication between keratinocytes. Previous studies found that PKCμ, a novel PKC isozyme, regulates efficient cutaneous wound healing. However, the molecular mechanism by which PKCμ regulates wound healing remains unknown. We have identified that PKCμ suppresses intercellular communication and enhances cell migration in an *in vitro* wound healing model by regulating Cx43 containing gap junctions. PKCμ can directly interact with and phosphorylate Cx43 at S368, which leads to Cx43 internalization and downregulation. Finally, utilizing phosphomimetic and non-phosphorylatable S368 substitutions and gap junction inhibitors, we confirmed that PKCμ regulates intercellular communication and *in vitro* wound healing by controlling Cx43-S368 phosphorylation. These results define PKCμ as a critical regulator of Cx43 phosphorylation to control cell migration and wound healing in keratinocytes.

## Highlights

• Tissue wounding *in vivo*, or PMA stimulation *in vitro*, activates PKCμ
• PKCμ directly interacts with and phosphorylates Cx43 at S368
• Cx43 phosphorylation by PKCμ suppresses intercellular communication
• Cell migration is enhanced by PKCμ-mediated phosphorylation of Cx43

## Graphical abstract

**Figure:**
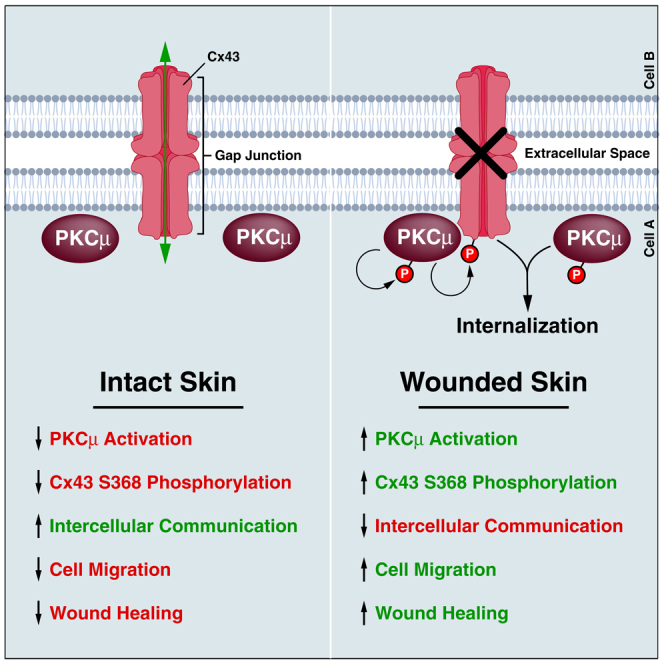

## Introduction

Wound healing is an intricate physiological response to an injury that encompasses four phases; hemostasis, inflammation, proliferation, and tissue remodeling.^1^ Complications in wound healing can arise at any stage and lead to chronic, or non-healing, wounds that have been well documented in wounds associated with diabetic foot ulcers, venous ulcers, surgery, and the aging population.^2^ Downregulation of intercellular communication between various cell types is essential for efficient wound healing. Direct communication between cells is mediated in part through gap junctions composed of connexin proteins which allow for the exchange of small ions and secondary metabolites between adjacent cells.^3^ Connexin 43 (Cx43) is the most abundantly expressed connexin isoform in the stratum basale and spinosum layers of the skin epidermis.^4^ In fact, Cx43 expression is dynamically regulated following wounding such that intercellular communication is dramatically reduced in the suprabasal cells near the wound edge.^5^^,^^6^^,^^7^ Wounded murine skin treated with topical Cx43 antisense RNA gel that reduces Cx43 expression enhances keratinocyte migration and reduces inflammation.^8^^,^^9^^,^^10^ Similarly, heterozygous Cx43 knockout mice display accelerated wound closure due to enhanced proliferation and re-epithelialization.^11^

Cx43 function is tightly regulated by multiple phosphorylation events in its intracellular carboxy-terminal domain.^12^ Phosphorylation of Cx43 at Serine-368 reduces channel conductance and promotes the degradation of Cx43 plaques through lysosomal and proteasomal degradation pathways, thereby reducing intercellular signaling.^13^^,^^14^ Cx43-S368 phosphorylation is also highly regulated during wound healing. The unwounded human epidermal layer contains an even distribution of Cx43 with low levels of S368 phosphorylation. In contrast, phosphorylated Cx43-S368 is significantly increased in basal keratinocytes 24 h post wounding. A key role for Cx43-S368 has been emphasized by the fact that treatment with Cx43 specific peptide inhibitor α-CT-1 accelerates wound healing *in vivo*.^15^ It has also been reported that α-CT-1 enhances the phosphorylation of Cx43-S368 in injury models including scratch wounded cultured cells and *in vivo* models of ventricular injury.^16^

A variety of kinases have been shown to target Cx43, including members of the Protein Kinase C (PKC) family which phosphorylate S368 on the C-terminal tail of Cx43.^14^^,^^17^ PKCs are a superfamily of serine/threonine kinases that play a vital role in multiple cell functions such as proliferation, migration, and apoptosis.^18^ There are three classes of PKCs based on their secondary messenger requirements. Classical PKCs (α, β_I_, β_II_, and γ) require diacylglycerols (DAGs) and calcium ions for activation whereas novel PKC isozymes (δ, ε, η, μ, and θ) only require DAGs. Atypical PKCs (ι and ζ), on the other hand, require neither DAG nor calcium ions for activation.^19^^,^^20^^,^^21^ Novel PKC isozymes PKCδ and PKCε can directly phosphorylate Cx43 at S368. This phosphorylation is known to alter the conductivity of the channel itself as well as signal the degradation of Cx43 plaques.^22^^,^^23^^,^^24^^,^^25^ Furthermore, both PKCδ and PKCε have been implicated in the regulation of wound healing.^26^^,^^27^

A recent study has implicated another novel PKC isozyme, PKCμ (PRKD1), in wound healing *in vivo*.^28^ Interestingly, among PKC isoforms, PKCμ (PRKD1) is thought to only play a minor role in regulating Cx43 as it was not observed to be downregulated following prolonged 12-O:-tetradecanoylphorbol-13-acetate (TPA) treatment as other PKC isoforms in rat R6 fibroblasts.^29^ PKCμ is a novel PKC isozyme that can be activated by DAG such as TPA or Phorbol 12-myristate 13-acetate (PMA), but does not require calcium, and plays a role in various cellular processes including cell growth, differentiation, and apoptosis.^30^ Mice with PKCμ knocked out specifically in the stratified epithelia display reduced keratinocyte proliferation, migration, and delayed wound healing.^28^ However, the molecular mechanism by which PKCμ regulates these cellular processes related to wound healing remains unknown.

Given that PKCμ has recently been shown to impact cutaneous skin wound healing and that PKCs play a role in regulating Cx43 led intercellular communication, we reasoned that PKCμ may target Cx43 to control efficient wound healing in the keratinocyte setting. Here, we describe PKCμ as a Cx43 interacting kinase that directly phosphorylates Cx43 at S368, promoting Cx43 internalization into the cytosol and its downregulation, thereby reducing intercellular communication to enhance cell migration in an *in vitro* wound healing assay. PKCμ is upregulated in cultured keratinocytes and human skin biopsies following wounding. Inhibition or depletion of PKCμ led to a decrease in Cx43-S368 phosphorylation in cultured keratinocytes along with a reduction in cell migration. However, the gap junction inhibitor Carbenoxolone significantly reversed the delayed wound healing phenotype. Furthermore, α-CT1, a Cx43 specific inhibitor, also accelerated wound healing in PKCμ inhibited or depleted keratinocytes. Based on these data, we propose that PKCμ enhances keratinocyte migration/wound healing through suppressing intercellular communication by controlling Cx43-S368 phosphorylation.

## Results

### Inhibition or depletion of PKCμ impairs cell migration

**Figure 1:**
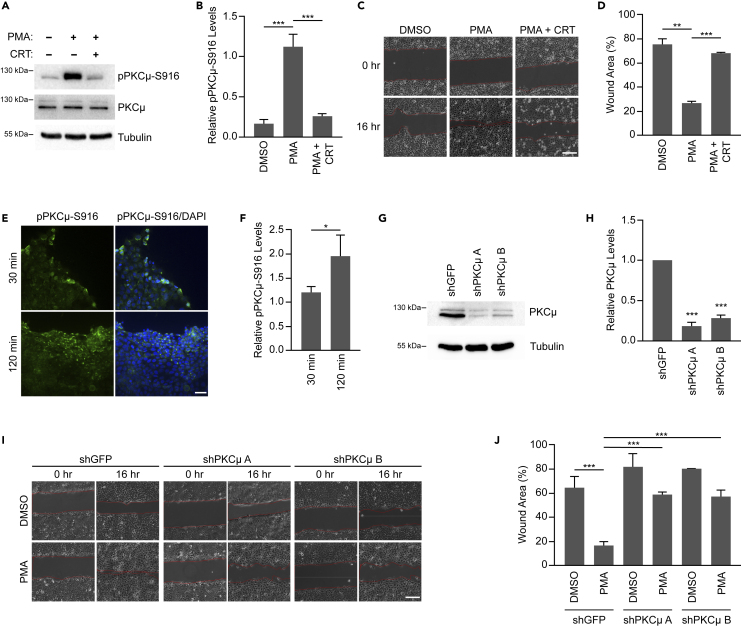
PKCμ inhibition/reduction impairs wound healing *in vitro* (A) HaCaT cells treated with PMA (10 nM) in the absence or presence of CRT (1 μM CRT) for 16 h. Western blots of cell lysates probed for pPKCμ-S916, PKCμ, and tubulin. (B) Relative pPKCμ-S916 levels in treated samples compared to DMSO control based on densitometric analysis from three replicate experiments as shown in (A). (C) Representative images of *in vitro* wound healing assay at 0 and 16 h on HaCaT cells treated with DMSO, PMA (10 nM), or PMA with CRT (1 μM CRT). Scale bar = 100 μm. (D) Percentage of wound area remaining after 16 h from three replicate experiments as shown in (C). (E) Representative images from immunofluorescence staining of pPKCμ-S916 in scratch wounded HaCaT cells at 30- and 120-min post-wounding. Scale bar = 40 μm. (F) Relative pPKCμ-S916 levels based on fluorescence intensity from three images from 30- and 120-min post-wounding as shown in (E). Relative levels are based on 10 random fields taken from cells adjacent to the wound and cells interior to the wound for each time point. (G) HaCaT cells transduced with control shRNA (shGFP) or two shRNAs directed against PKCμ. Cell lysates were probed by western blotting for PKCμ and tubulin. (H) Quantification of PKCμ levels relative to shGFP from three replicate experiments as shown in (G). (I) Representative images of *in vitro* wound healing assay at 0 and 16 h on HaCaT cells transduced with control shRNA (shGFP) or two shRNAs directed against PKCμ treated in the absence or presence of PMA (10 nM). Scale bar = 100 μm. (J) Percentage of wound area remaining after 16 h from three replicate experiments as shown in (I). All calculations are based on three replicates −/+ S.D. ∗∗p < 0.01, ∗∗∗p < 0.001 (Student’s *t* test).

We first examined the effect of PKCμ inhibition on wound healing in HaCaT immortalized human keratinocytes. The DAG mimetic PMA is well established to promote epithelial cell migration.^31^ PMA is also known to bind to the C1 domain within the N-terminal regulatory region of PKCμ leading to its translocation to the plasma membrane.^32^^,^^33^^,^^34^ In addition, PMA activates PKCμ upon which PKCμ autophosphorylates S916 within its C-terminal tail, which also serves as a marker of PKCμ activation.^35^ To assess the ability of PMA to activate PKCμ in HaCaT cells, we incubated cells with PMA in the absence or presence of the PKCμ specific inhibitor CRT 0066101 dihydrochloride (CRT). We found that PMA induced robust activation of PKCμ as shown by S916 phosphorylation, which was completely reversed by CRT treatment (Figures 1A and 1B). To assess whether PKCμ activity is required for cell migration in an *in vitro* wound healing assay, we treated a monolayer of HaCaT cells with PMA in the absence or presence of CRT followed by inducing a scratch in the monolayer and monitored wound closure after 16 h. While we observed that PMA treatment dramatically accelerated wound healing, PKCμ inhibition by CRT suppressed PMA induced wound healing in HaCaT cells (Figures 1C and 1D). CRT treatment in the absence of PMA did not regulate HaCaT cell migration (Figures S1A and S1B). To determine if an increase in PKCμ activation was also observed during monolayer wounding, we stained HaCaT cells for pPKCμ-S916 at 30- and 120-min following scratch administration. In this setting, similar to PMA treatment, we observed an increase in pPKCμ-S916 staining in cells adjacent to the wound compared to cells away from the wound, which increased with time (Figures 1E and 1F). These data suggests that PKCμ is activated upon wounding and promotes cell migration in an *in vitro* model of wound healing.

PKCμ has three isoforms namely PRKD1, PRKD2, and PRKD3 which can all be activated by DAGs such as PMA.^36^ Since CRT inhibits all isoforms of PKCμ,^37^^,^^38^ we utilized shRNA to specifically deplete PKCμ (PRKD1) in HaCaT cells (Figures 1G, 1H, and S1C). Following depletion of PKCμ with two independent shRNAs, an *in vitro* wound healing assay was conducted in the absence or presence of PMA. Depletion of PKCμ in HaCaT cells dramatically delayed wound closure in the presence of PMA (Figures 1I and 1J). Overall, these results demonstrate that PKCμ is required for PMA-induced keratinocyte cell migration *in vitro*, suggesting that the delay in wound healing in PKCμ knockout mice may be through reduced keratinocyte migration.^28^

In our *in vitro* wound healing assays, we treated our cultures with mitomycin C prior to scrape initiation to inhibit cell proliferation such that the observed wound closure was solely due to cell migration. A previous study demonstrated that mitomycin C treatment promoted an increase in Cx43 levels and Cx43-mediated intercellular communication in corneal endothelial cells.^39^ To demonstrate that changes in cell migration in our HaCaT cells were not due to mitomycin C regulation of Cx43-mediated intercellular communication, we carried out a scrape loading/dye transfer (SL/DT) assay through which the fluorescent dye Lucifer yellow was introduced into cells through a scalpel-induced incision in the monolayer and its transfer into contiguous cells, which is dependent on gap junctions, was monitored.^40^ We found that mitomycin C treatment did not influence Cx43 protein levels or Cx43-dependent intercellular communication occurring through gap junctions (Figures S1D–S1F). In addition, PKCμ-mediated regulation of wound healing was also observed when serum starvation was used to suppress cell division (Figures S1G and S1H) or under untreated conditions (Figures S1I and S1J). These results demonstrate that mitomycin C does not appear to influence Cx43-mediate intercellular communication in HaCaT cells as it may in corneal endothelial cells as previously observed.^39^

### PKCμ phosphorylates Cx43 at Serine-368 to regulate intercellular communication

**Figure 2:**
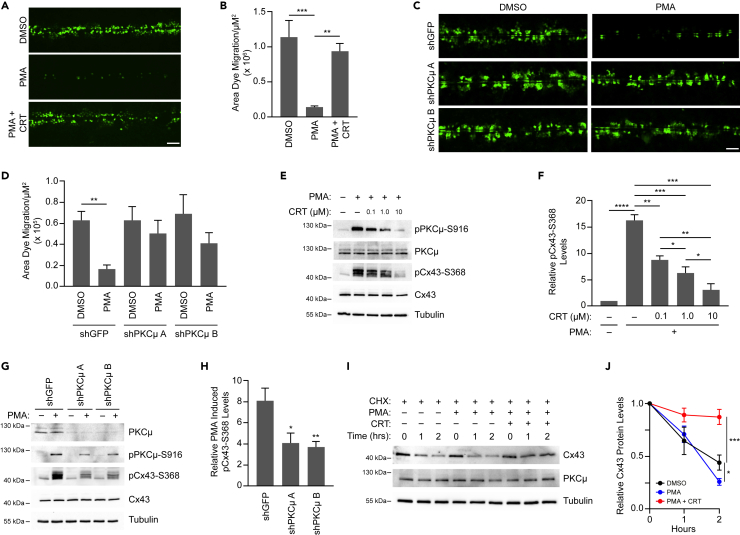
PKCμ regulates the level of intercellular communication and Cx43-S368 phosphorylation (A) Representative fluorescent microscopic images of SL/DT assay in HaCaT cells treated with CRT (5 μM) for 6 h followed by PMA (0.5 μM) for 30 min. Scale bar = 100 μm. (B) Quantification of dye migration area from three replicate experiments as shown in (A). (C) Representative fluorescent microscopic images of SL/DT assay in HaCaT cells transduced with control shRNA (shGFP) or two shRNAs directed against PKCμ and treated with or without PMA (0.5 μM) for 30 min. Scale bar = 100 μm. (D) Quantification of dye migration area from three replicate experiments as shown in (C). (E) HaCaT cells treated with CRT at indicated concentrations for 6 h followed by a 30-min treatment with PMA (0.5 μM). Cell lysates were probed by western blotting for pPKCμ-S916, PKCμ, pCx43-S368, Cx43, and tubulin. (F) Quantification of pCx43-S368 in indicated treated conditions compared to DMSO based on densitometric analysis from three replicate experiments as shown in (E). (G) HaCaT cells transduced with control shRNA (shGFP) or two shRNAs directed against PKCμ and treated with or without PMA (0.5 μM) for 30 min. Cell lysates were probed by western blotting for pPKCμ-S916, PKCμ, pCx43-S368, Cx43, and tubulin. (H) Quantification of pCx43-S368 in indicated treated conditions compared to DMSO based on densitometric analysis from three replicate experiments as shown in (G). (I) HaCaT cells were treated with Cycloheximide (100 μg/mL) in the absence or presence of PMA (0.5 μM) with or without CRT (5 μM). Whole-cell lysates were collected at indicated time points and treated with Lambda phosphatase and subsequently probed by western blotting for Cx43, PKCμ, and tubulin. (J) Relative levels of total Cx43 for each treatment group compared to T = 0 h. All calculations are based on three replicates −/+ S.D. ∗p < 0.05, ∗∗p < 0.01, ∗∗∗p < 0.001 (Student’s *t* test).

Gap junctions composed of Cx43 are prevalent in keratinocytes, and intercellular communication through these gap junctions decreases dramatically along the wound edge *in vivo*.^7^ Similarly, Cx43 levels are significantly reduced in the suprabasal keratinocytes during wound healing.^41^ To determine whether PKCμ inhibition or depletion regulates intercellular communication, we conducted SL/DT assays in HaCaT cells treated with PMA in the absence or presence of CRT. Consistent with our results observed in the *in vitro* wound healing assay, we found that PMA dramatically suppressed intercellular communication, which was substantially blocked when PKCμ was inhibited by CRT (Figures 2A and 2B). CRT alone did not influence intercellular communication in the SL/DT assay (Figures S2A and S2B). Furthermore, the PMA-induced suppression of intercellular communication was also reversed when HaCaT cells were depleted of PKCμ by shRNA, as PKCμ depleted cells displayed high levels of dye migration even in the presence of PMA (Figures 2C and 2D). These data suggest that PKCμ is an important mediator of gap junction-mediated intercellular communication in response to PMA.

Phosphorylation of Cx43 on S368 reduces channel conductance and signals Cx43 plaque internalization and degradation.^14^ Therefore, we next assessed whether suppressing PKCμ activity influences Cx43-S368 phosphorylation status. When HaCaT cells were treated with PMA, the phosphorylation level of Cx43 was dramatically enhanced, which was reversed by the PKCμ inhibitor CRT in a dose-dependent manner (Figures 2E and 2F). Furthermore, the decrease in Cx43-S368 phosphorylation status due to CRT treatment mirrored that of PKCμ-S916, indicating a direct correlation between PKCμ activation status and Cx43-S368 phosphorylation (Figures 2E, 2F, and S2C). Similarly, HaCaT cells depleted of PKCμ also showed reduced levels of pCx43-S368 compared to control shRNA treated cells in the presence of PMA, which again mirrored the decrease in PMA induced PKCμ activation status (Figures 2G and 2H). Similar results were also observed in mouse embryonic fibroblasts (MEFs) that were either treated with the PKCμ inhibitor CRT or depleted of PKCμ with shRNA, suggesting a potentially wider relevance of this signaling pathway (Figures S2D and S2E). To determine if downregulation of Cx43, another consequence of S368 phosphorylation, was influenced by PKCμ inhibition, HaCaT cells were treated in the presence of PMA with or without CRT along with cycloheximide to inhibit *de novo* protein synthesis.^42^ Monitoring the loss of Cx43 following cycloheximide, we found that PMA treatment enhanced degradation of Cx43, whereas treating with CRT blocked the PMA-induced degradation of Cx43 (Figures 2I and 2J). Taken together, these results suggest that Cx43-S368 phosphorylation and degradation are regulated by PKCμ.

### PKCμ colocalizes with Cx43

**Figure 3:**
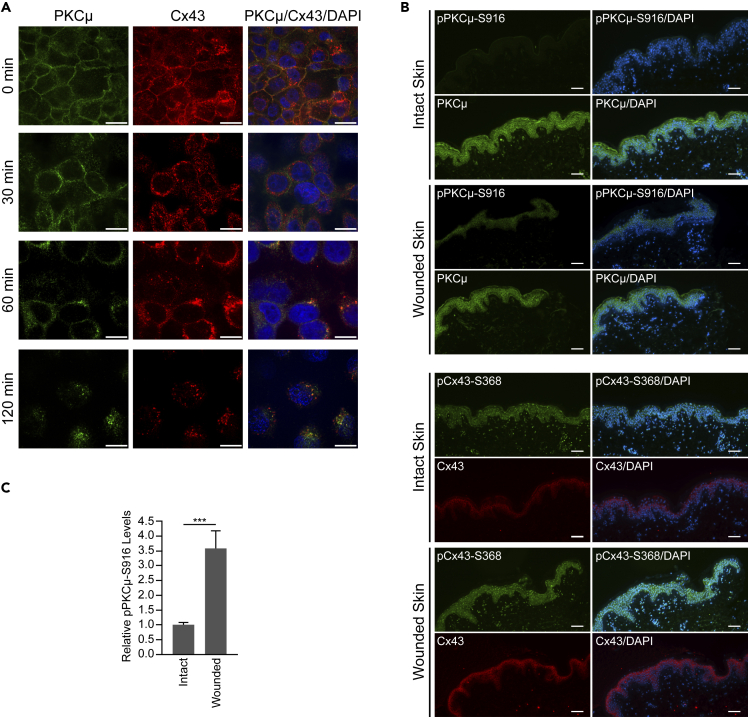
PKCμ and Cx43 colocalize in HaCaT cells (A) Representative images of immunofluorescence staining of PKCμ (green) and Cx43 (red) in HaCaT cells treated with PMA at different time points. Scale bar = 10 μm. (B) Immunofluorescence images of intact and wounded human skin stained for pPKCμ-S916, total PKCμ, pCx43-S368, and total Cx43. Scale bar = 100 μm. (C) Relative pPKCμ-S916 levels in intact and wounded skin based on immunofluorescence signal intensity of images shown in (B). Error bars represent S.D. ∗∗∗p < 0.001 (Student’s *t* test).

Next, we conducted immunofluorescence staining to determine the subcellular localization of PKCμ and Cx43 in the presence or absence of PMA. We observed that PKCμ and Cx43 colocalized near the plasma membrane (Figure S3A). Furthermore, upon PMA stimulation, both PKCμ and Cx43 appeared to be internalized from the plasma membrane and continued to colocalize in the cytosol (Figure S3A). Following a time course of PMA treatment, we found that PKCμ and Cx43 showed colocalization in untreated cells at the membrane and further co-localization in internalized regions in the cytosol (Figure 3A). Interestingly, at 30 min post PMA treatment, the two signals appeared to be separated, with PKCμ remaining closer to the interface of adjacent cells than Cx43. These results indicate that while PKCμ and Cx43 function at similar locations in the cell and are trafficked from the plasma membrane similarly following PMA stimulation, the dynamics of internalization of the two proteins may differ, suggesting that the interaction between PKCμ and Cx43 may be transient and that the two proteins likely do not form a long-lasting stable complex. Furthermore, when human skin samples were either left unwounded or subjected to a punch biopsy wound, we observed that phosphorylation of PKCμ-S916 increased 3.6-fold in the epidermis (Figures 3B and 3C). In addition, in these human skin biopsies, pCx43-S368 appears to be internalized into the cytosol and localized in a perinuclear fashion, similar to the localization of Cx43 in HaCaT cells following treatment with PMA (Figure 3A). Taken together, these data support the notion that stimulation of human keratinocyte-derived cells or human skin tissue, either through PMA treatment or wounding, promotes PKCμ-S916 phosphorylation and subsequent Cx43-S368 phosphorylation and internalization, to suppress intercellular communication to promote wound healing.

### PKCμ interacts with, and phosphorylates, Cx43 at Serine-368 *in vitro*

**Figure 4:**
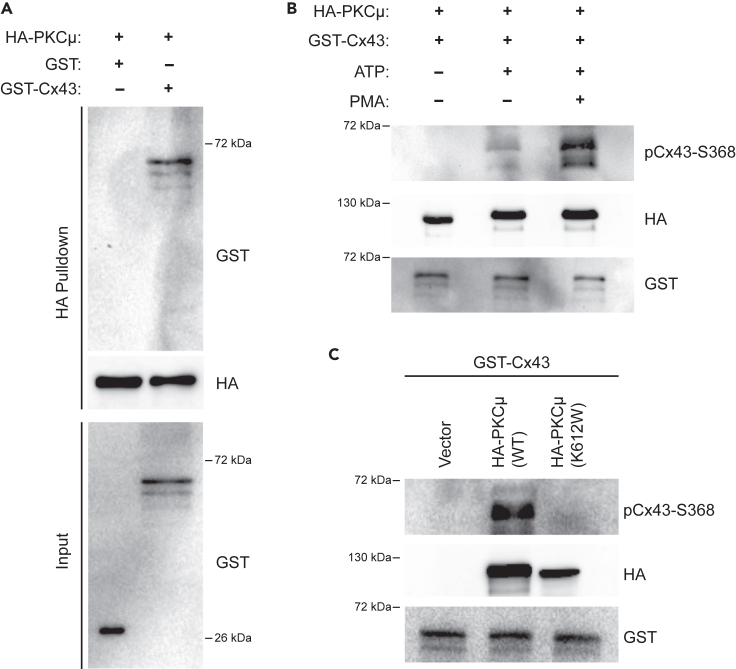
PKCμ interacts with Cx43 and phosphorylates Cx43 at S368 (A) *In vitro* pull-down assay with HA-PKCμ attached to HA antibody conjugated agarose beads were incubated with either GST or GST-Cx43 and subsequently washed to remove unbound material. Pulled down material was probed by western blotting for GST and HA. (B) *In vitro* kinase assay with HA-PKCμ immunoprecipitated from untreated or PMA treated transfected HEK 293T cells incubated with bacterially expressed GST-Cx43 in the presence or absence of ATP (200 μM). Reactions were probed by western blotting for pCx43-S368, HA, and GST. (C) *In vitro* kinase assay as described in (B) with wild-type (WT) or catalytically inactive (K612W) HA-PKCμ and GST-Cx43. Reactions were probed by western blotting for pCx43-S368, HA, and GST.

Prior studies have shown that PKCδ and PKCε isozymes interact with, and phosphorylate, Cx43 at S368.^13^^,^^43^ The C-terminal tail of Cx43 consists of a PKC consensus sequence (RXSSR) corresponding to the amino acid sequence RASSR in which the first serine residue is S368.^44^ Since we have shown that PKCμ inhibition or depletion reduces Cx43-S368 phosphorylation, we examined whether PKCμ directly interacts with Cx43. To this end, we purified glutathione S-transferase (GST) fused to the amino-terminus of full-length Cx43 (GST-Cx43), as well as unfused GST (serving as a negative control) (Figures S3B and S3C). Amino-terminal hemagglutinin (HA) epitope-tagged PKCμ (HA-PKCμ) was immunoprecipitated with HA antibody conjugated to agarose from transfected HEK 293T cells. Interestingly, HA-PKCμ transfected 293T cells had a higher basal level of Cx43-S368 phosphorylation when compared to cells transfected with the control vector (Figure S3D), corroborating our finding that Cx43-S368 phosphorylation is controlled, in part, by PKCμ. Utilizing immunoprecipitated HA-PKCμ that remained bound to agarose beads, we performed an *in vitro* pull-down assay in the presence of GST or GST-Cx43. We found that GST-Cx43 bound HA-PKCμ, whereas untagged GST did not (Figure 4A), indicating that PKCμ is capable of directly interacting with Cx43 *in vitro*.

PKCμ targets multiple substrates for phosphorylation to regulate downstream cellular functions.^45^ While our studies above indicate that PKCμ can regulate Cx43-S368 phosphorylation in cells, to examine whether PKCμ can directly phosphorylate Cx43-S368, we conducted an *in vitro* kinase assay with HA-PKCμ and GST-Cx43 in the presence or absence of ATP. First, we observed a phosphorylated S368 signal in GST-Cx43 when incubated with HA-PKCμ in an ATP-dependent manner (Figure 4B). Furthermore, when we purified HA-PKCμ from PMA treated cells, we observed an enhanced ability of HA-PKCμ to phosphorylate GST-Cx43 on S368 (Figure 4B), suggesting that purified HA-PKCμ from PMA treated cells had a higher activation status. To ensure that the kinase activity of HA-PKCμ immunoprecipitated material was due to HA-PKCμ and was not contributed by a co-purifying enzyme, we transfected 293T cells with wild-type HA-PKCμ and a catalytically inactive version of HA-PKCμ (K612W) which harbors a mutation in the ATP-binding site of PKCμ.^46^^,^^47^^,^^48^ Each of these HA-PKCμ proteins were immunoprecipitated and utilized in an *in vitro* kinase assay. While we found that wild-type HA-PKCμ was efficient at phosphorylating GST-Cx43, catalytically inactive HA-PKCμ K612W was unable to carry out this activity (Figure 4C). Overall, these data suggest that PKCμ can directly bind to and phosphorylate Cx43 at S368.

### Inhibition of intercellular communication accelerates wound healing in PKCμ inhibited or depleted cells

**Figure 5:**
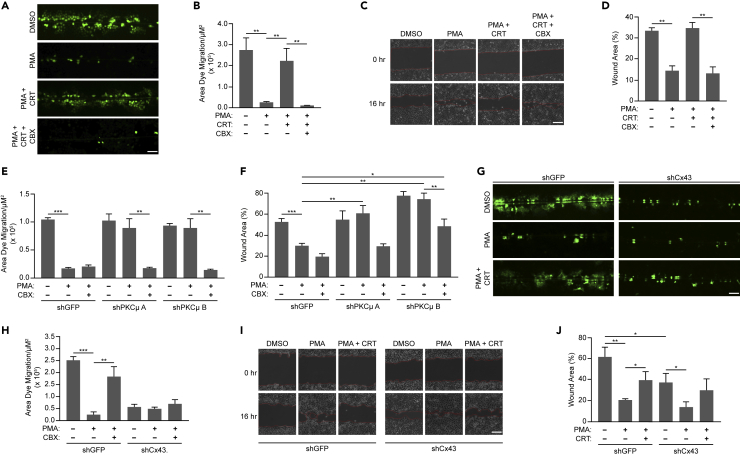
PKCμ regulates wound healing through gap junction mediated intercellular communication (A) Representative fluorescent microscopic images of SL/DT assay in HaCaT cells treated in the absence or presence of CRT (1 μM CRT), with or without CBX (100 μM) for 6 h, followed by PMA (0.5 μM) for 30 min. Scale bar = 100 μm. (B) Quantification of dye migration area from three replicate experiments as shown in (A). (C) Representative images of *in vitro* wound healing assay in HaCaT cells treated with PMA (10 nM) in the presence or absence of CRT (1 μM), with or without CBX (50 μM) at 0 and 16 h. Scale bar = 100 μm. (D) Percentage of wound area remaining after 16 h from three replicate experiments in (C). (E) Quantification of dye migration area from SL/DT assays in HaCaT cells expressing shGFP, shPKCμ A, or shPKCμ B, and treated with or without CBX (100 μM) for 6 h followed by PMA (0.5 μM) for 30 min from three replicate experiments in (Figure S4A). (F) Percentage of wound area remaining after 16 h in HaCaT cells expressing with shGFP, shPKCμ A, or shPKCμ B, in the absence or presence of PMA (0.5 μM), with or without CBX (100 μM) for 16 h from three replicate experiments in (Figure S4B). (G) Representative fluorescent microscopic images of SL/DT assay in HaCaT cells expressing shGFP or shCx43 and treated in the absence or presence of CRT (1 μM) for 5 h with or without PMA (0.5 μM) for 30 min. Scale bar = 100 μm. (H) Quantification of dye migration area from three replicate experiments as shown in (G). (I) Representative images of *in vitro* wound healing assay of shGFP or shCx43 treated HaCaT cells treated with PMA (10 nM) in the presence or absence of CRT (1 μM) at 0 and 16 h. Scale bar = 100 μm. (J) Percentage of wound area remaining after 16 h from three replicate experiments shown in (I). All calculations are based on three replicates −/+ S.D. ∗p < 0.05, ∗∗p < 0.01, ∗∗∗p < 0.001 (Student’s *t* test).

To investigate whether PKCμ regulates *in vitro* wound healing through Cx43-containing gap junctions, we blocked gap junction activity with the non-selective inhibitor Carbenoxolone (CBX) which is a glycyrrhetinic acid derivative that uncouples gap junctions.^49^ We first set out to determine whether the reversal of PMA-mediated suppression of intercellular communication by the PKCμ inhibitor CRT was dependent on gap junction conductance. To this end, we cotreated HaCaT cells with PMA and CRT, in the absence or presence of CBX, followed by SL/DT assays. We observed that CBX inhibition dramatically reduced intercellular communication in cells treated with PMA and CRT (Figures 5A and 5B). Next, utilizing the same treatments, we assessed whether cell migration regulated by PMA and CRT in an *in vitro* wound healing assay would be similarly influenced by the gap junction inhibitor. While PMA treatment accelerated wound closure, which was blocked by CRT, we observed that treatment with CBX reversed the effect of CRT and enhanced wound healing to a similar extent as PMA treatment alone, suggesting that gap junction inhibition plays a key role in cell migration and wound healing regulated by PKCμ (Figures 5C and 5D). In addition, we conducted SL/DT and *in vitro* wound healing assays in control and PKCμ-depleted HaCaT cells treated with or without CBX. PMA dramatically reduced intercellular communication of HaCaT cells, which was blocked when PKCμ was depleted (Figures 5E and S4A). Furthermore, CBX treatment completely reversed the effect of PKCμ depletion on PMA-induced suppression of intercellular communication (Figures 5E and S4A). Similarly, *in vitro* wound healing assays showed that gap junction inhibition with CBX increased the rate of wound healing in PKCμ-depleted HaCaT cells (Figures 5F and S4B). Next, we assessed whether depleting Cx43 would block the effect of PKCμ inhibition on intercellular communication. To this end, we generated HaCaT cells depleted of Cx43 using shRNA (Figure S5A). Control and Cx43-depleted cells were then treated with PMA in the absence or presence of CRT, and intercellular communication was measured by SL/DT assay. We found that PMA suppressed intercellular communication, which was reversed by CRT in control cells (Figures 5G and 5H). However, Cx43-depleted cells treated with PMA showed reduced intercellular communication similar to control cells treated with PMA, but CRT was unable to reverse the PMA-induced suppression of intercellular communication (Figures 5G and 5H). In an *in vitro* wound healing assay, we observed that basal cell migration was enhanced in Cx43-depleted cells compared to control cells and CRT was unable to reverse the effects of PMA treatment on cell migration (Figures 5I and 5J). Overall, these results indicated that PKCμ regulation of cell migration in an *in vitro* wound healing assay is mediated by PKCμ controlling intercellular communication through Cx43 containing gap junctions.

### PKCμ-mediated suppression of intercellular communication and promotion of wound healing is through Cx43-S368 phosphorylation

**Figure 6:**
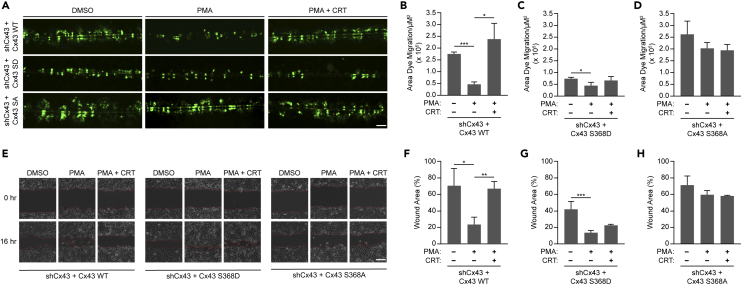
PKCμ regulates Cx43 led intercellular communication and wound healing through Cx43-S368 residue (A) Representative fluorescent images of SL/DT assay conducted in shCx43 expressing HaCaT cells reconstituted with either wild-type Cx43 (WT), or mutant versions of Cx43 (S368D (SD) or S368A (SA)) treated in the absence or presence of CRT (1 μM CRT) followed by PMA (0.5 μM) for 30 min. Scale bar = 100 μm. (B–D) Quantification of dye migration area in Cx43 WT (B), Cx43 S368D (C), and Cx43 S368A (D) reconstituted cells from three replicate experiments as shown in (A). (E) Representative images of *in vitro* wound healing assay of shCx43 expressing HaCaT cells reconstituted with either wild-type Cx43 (WT), or mutant versions of Cx43 (S368D or S368A) treated with PMA (10 nM) in the presence or absence of CRT (1 μM) at 0 and 16 h. Scale bar = 100 μm. (F–H) Percentage of wound area remaining in Cx43 WT (F), Cx43 S368D (G), and Cx43 S368A (H) reconstituted cells after 16 h from three replicate experiments as shown in (E). All calculations are based on three replicates −/+ S.D. ∗p < 0.05, ∗∗p < 0.01, ∗∗∗p < 0.001 (Student’s *t* test).

While we have shown that PKCμ suppresses intercellular communication and promotes wound healing in a manner that depends on functional gap junctions, and that this activity correlates with the regulation of Cx43-S368 phosphorylation, it remains unclear whether PKCμ-mediated phosphorylation of Cx43 on S368 serves as the molecular basis for PKCμ suppressing intercellular communication and promoting wound healing. To investigate whether PKCμ regulates Cx43-S368 phosphorylation to suppress intercellular communication and promote wound healing, we reconstituted Cx43-depleted HaCaT cells (Figure S5A) with exogenously expressed wild-type Cx43 (WT) or Cx43 carrying phosphorylation mimetic or non-phosphorylatable versions of S368 (S368D or S368A, respectively) (Figure S5B). Cx43-depleted cells suppressed intercellular communication and promoted wound healing to a greater extent than shGFP control cells (Figures 5G–5J), which is in accordance with prior studies demonstrating a role for Cx43-mediated intercellular communication and wound healing.^50^^,^^51^ Cx43-depleted HaCaT cells reconstituted with Cx43 WT regained basal intercellular communication, wound healing capacity, and responsiveness to CRT treatment in both SL/DT and *in vitro* wound healing assays (Figures 6A, 6B, 6E, and 6F). Cx43-depleted HaCaT cells reconstituted with the phosphomimetic Cx43 S368D phenocopied Cx43-depleted cells, showed reduced basal intercellular communication along with enhanced wound healing, and were unaffected by CRT treatment (Figures 6A, 6C, 6E, and 6G). In contrast, reconstitution with non-phosphorylatable Cx43 S368A did not show reduced intercellular communication or enhanced wound healing in response to PMA treatment, consistent with an inability to be phosphorylated at S368 to downregulate Cx43 protein levels and reduce intercellular communication (Figures 6A, 6D, 6E, and 6H). In addition, cells reconstituted with Cx43 S368A also showed no further changes in intercellular communication and cell migration in response to CRT treatment (Figures 6A, 6D, 6E, and 6H). These results indicate that the ability of PMA to suppress intercellular communication and promote wound healing in a PKCμ-dependent manner relies on Cx43 S368 phosphorylation.

### α-CT1 enhances S368 phosphorylation and increases the rate of *in vitro* wound healing in the absence of PKCμ

**Figure 7:**
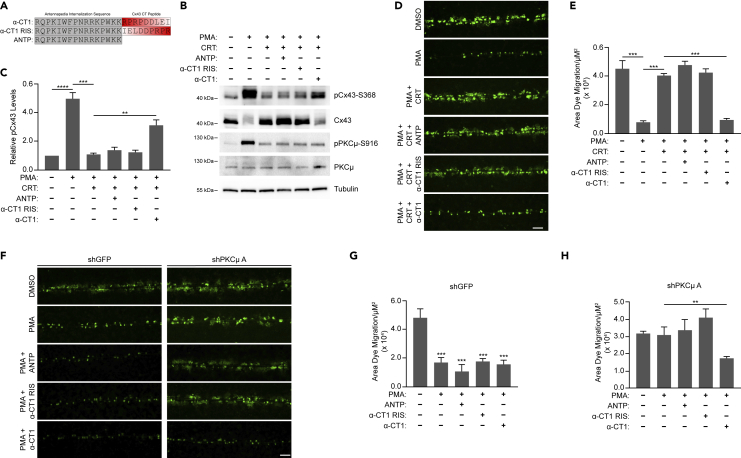
Cx43 specific inhibitor α-CT1 reduces intercellular communication in PKCμ inhibited/depleted HaCaT cells (A) Amino acid sequence of peptides α-CT1, reverse inactive peptide (α-CT1 RIS), and antennapedia control (ANTP). (B) HaCaT cells were scratched with a pipette tip followed by treatment with PMA (10 nM) with or without CRT (5 μM), in the absence or presence of ANTP, α-CT1 RIS, or α-CT1 peptides (each at 100 μM) for 16 h. Cell lysates were probed by western blotting for pCx43-S368 Cx43, pPKCμ-S916, PKCμ, and tubulin. (C) Relative pCx43-S368 levels in treated samples compared to DMSO control based on densitometric analysis from three replicate experiments as shown in (A). (D) Representative fluorescent microscopic images of SL/DT assay in HaCaT cells treatment with or without CRT (5 μM), in the absence or presence of ANTP, α-CT1 RIS, or α-CT1 peptides (each at 100 μM) for 6 h followed by PMA (10 nM) for 30 min. Scale bar = 100 μM. (E) Quantification of dye migration area from three replicate experiments as shown in (D). (F) Representative fluorescent microscopic images of SL/DT assay in HaCaT cells transduced with control shRNA (shGFP) or an shRNA directed against PKCμ and in the absence or presence of ANTP, α-CT1 RIS, or α-CT1 peptides (each at 100 μM) for 6 h followed by PMA (10nM) for 30 min. Scale bar = 100 μm. (G and H) Quantification of dye migration area from three replicate experiments as shown in (F) for shGFP (G) or shPKCμ. (H) All calculations are based on three replicates −/+ S.D. ∗∗p < 0.01, ∗∗∗p < 0.001 (Student’s *t* test).

CBX is a non-selective gap junction inhibitor that can inhibit multiple connexins expressed in keratinocytes such as Cx26 and Cx43.^52^ Therefore, we next utilized a Cx43 specific inhibitor termed alpha-carboxyl terminus 1 (α-CT1) which is a 25 amino acid peptide comprising the antennapedia internalization sequence and the carboxyl-terminal tail of Cx43.^53^ The mechanism of action of α-CT1 is that it inhibits the interaction between the tight junction associated protein Zonula Occludens 1 (ZO-1) and the C-terminus of Cx43.^54^ Previous studies have shown that treatment with α-CT1 in either mouse cardiac injury models or scratch wounded HeLa cells increases S368 phosphorylation of Cx43.^16^ Since our previous data demonstrated that Cx43-S368 phosphorylation is lower in PCKμ inhibited HaCaT cells, we examined if α-CT1 can increase Cx43-S368 phosphorylation in an *in vitro* wound healing assay in HaCaT cells. In addition to α-CT1, we utilized the antennapedia internalization sequence alone (ANTP) and reverse inactive sequence of α-CT1 (α-CT1 RIS) as control peptides (Figure 7A). HaCaT cells were treated with PMA in the absence or presence of CRT with or without ANTP, α-CT1 RIS, or α-CT1, subjected to a scratch, and incubated for 16 h before preparing cell lysates. Western blotting assays showed that α-CT1 treatment increased phosphorylated Cx43-S368 in scratch wounded HaCaT cells treated with a combination of PMA and the PKCμ inhibitor CRT (Figures 7B and 7C). Interestingly, total Cx43 protein levels were dramatically lower in the α-CT1 treated group similar to the cells treated with PMA alone, suggesting that PMA-dependent phosphorylation of Cx43-S368, which was suppressed by PKCμ inhibition/depletion, was reversed by α-CT1 treatment. Interestingly, this loss of Cx43 over the 16-h time course, which is blocked by CRT, is consistent with analysis of Cx43 protein stability observed when PMA destabilizes Cx43 which is blocked by CRT (Figures 2I and 2J), further supporting the notion that PKCμ is a critical regulator of Cx43 stability in keratinocytes.

**Figure 8:**
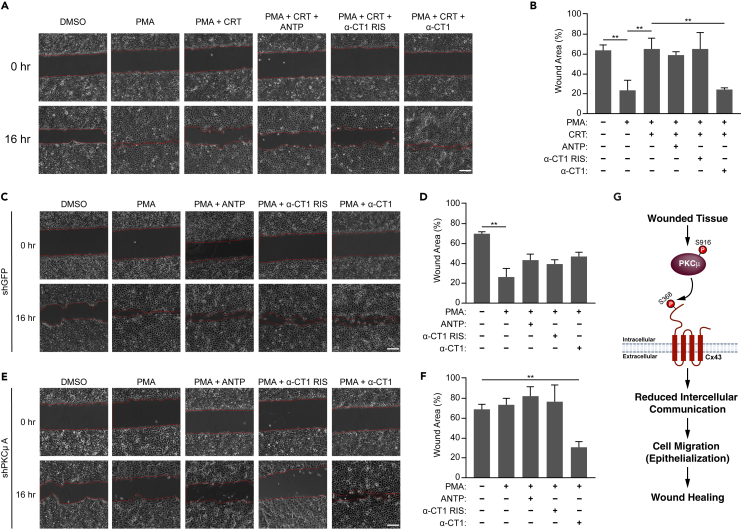
Cx43 Specific inhibitor α-CT1 accelerates wound healing in PKCμ inhibited/depleted HaCaT cells (A) Representative images of *in vitro* wound healing assay at 0 and 16 h in HaCaT cells treated with PMA (10 nM) with or without CRT (5 μM), in the absence or presence of ANTP, α-CT1 RIS, or α-CT1 peptides (each at 100 μM). Scale bar = 100 μm. (B) Percentage of wound area remaining after 16 h from three replicate experiments as shown in (A). (C–F) Representative images of *in vitro* wound healing assay at 0 and 16 h in HaCaT cells transduced with control shRNA (shGFP) (C) or an shRNA directed against PKCμ (E) treated with PMA (10 nM) in the absence or presence of ANTP, α-CT1 RIS, or α-CT1 peptides (each at 100 μM). Scale bar = 100 μm. Percentage of wound area remaining after 16 h from three replicate experiments in cells transduced with shGFP (D) as shown in (C), or cells transduced with shPKCμ (F) as shown in (E). (G) Schematic depicting PKCμ regulating Cx43-S368 phosphorylation to suppress intercellular communication and promote wound healing. All calculations are based on three replicates −/+ S.D. ∗∗p < 0.01 (Student’s *t* test).

Given that α-CT1 induced the phosphorylation of Cx43 S368 even in the presence of PMA/CRT, α-CT1 may promote Cx43 phosphorylation in a manner independent of PKCμ. Next, we conducted SL/DT assays to determine the effect of α-CT1 on intercellular communication in PKCμ inhibited cells. Treatment with control peptides (ANTP or α-CT1 RIS) did not influence the level of intercellular communication. However, α-CT1 treatment significantly reduced intercellular communication in HaCaT cells in which PKCμ was inhibited by CRT (Figures 7D and 7E). We also carried out SL/DT assays in PKCμ-depleted HaCaT cells and observed that α-CT1 treatment significantly reduced intercellular communication in PKCμ depleted cells (Figures 7F–7H, S6A, and S6B). In addition to SL/DT assays, we carried out *in vitro* wound healing assays to determine whether α-CT1 can rescue the delayed wound healing observed in PKCμ inhibited or depleted HaCaT cells. In assays using CRT to inhibit PKCμ, α-CT1 treatment significantly increased the rate of cell migration and *in vitro* wound healing (Figures 8A and 8B). Similarly, α-CT1 treatment increased cell migration in PKCμ-depleted HaCaT cells (Figures 8C–8F, S7A, and S7B). Given α-CT1 induced the phosphorylation of Cx43 S368 independent of PKCμ and reversed the effects of PKCμ inhibition or loss, these results indicate that PKCμ regulates cell migration and *in vitro* wound healing by regulating the phosphorylation status of Cx43-S368 to control gap junction function and intercellular communication (Figure 7G).

## Discussion

Efficient cutaneous wound healing requires suppression of intercellular communication which is achieved by increasing Cx43-S368 phosphorylation and downregulation of Cx43 containing gap junctions.^7^ On the other hand, delayed wound healing is accompanied by upregulation of the gap junction protein Cx43. Since gap junctional channel conductance and turnover rate of Cx43 are regulated by post-translational modifications of the Cx43 C-terminal tail, kinases that phosphorylate Cx43 may be viable targets for enhancing wound healing capacity, especially in clinical cases of chronic wounds.^12^ Previous studies have implicated novel PKC isozymes δ and ε in wound healing in human and mouse fibroblasts, respectively.^26^^,^^27^ Both PKC isozymes can phosphorylate Cx43 at S368.^13^^,^^43^ A recent study has discovered the role of another novel PKC isozyme termed PKCμ in cutaneous wound healing *in vivo*.^28^ However, the mechanism by which PKCμ regulates wound healing in the skin, and if it occurs through Cx43, is yet to be elucidated.

In the present study, we identified a previously unknown signaling pathway by which PKCμ regulates cell migration and *in vitro* wound healing by controlling intercellular communication mediated by the gap junction protein Cx43. Our data revealed that PKCμ regulates Cx43 by phosphorylating its C-terminal tail residue S368 (Figure 8G). Our *in vitro* studies suggested that PKCμ can directly bind and phosphorylate Cx43. Furthermore, immunofluorescence followed by confocal microscopy and colocalization analysis suggested that PKCμ and Cx43 co-localize at the plasma membrane. Interestingly, both PKCμ and Cx43 internalize into the cytosol upon treatment with PMA and continue to colocalize in these internalized structures. The dynamics of internalization appear to be different between PKCμ and Cx43, as PKCμ either remains on the membrane or is closer to the adjacent cell than Cx43 at an intermediate time point (Figure 3A, 30 min). In addition, Cx43 proteins that do not co-localize with PKCμ appear to form larger, more well-developed junctions, suggesting that the increased stability of Cx43 when not associated with PKCμ may protect these channels from being internalized. Thus, future studies on the internalization dynamics of both these proteins are warranted. Our data also showed extensive PKCμ staining outside of Cx43 positive regions indicating that while PKCμ may colocalize with Cx43 to promote its phosphorylation and downregulation, there are likely other functions of PKCμ outside of Cx43-containing gap junctions, and whether those other functions regulate intercellular communication and/or wound healing remains to be determined. In addition, we observed that in wounded human skin samples, PKCμ-S916 becomes hyperphosphorylated (Figure 3). Interestingly, we observed a much more pronounced increase in PKCμ-S916 phosphorylation compared to Cx43-S368. However, this may be due to the fact that we did not have control over when the wound would be administered in the human skin biopsy, and thus the tissue fixation may not have been carried out at an optimal time point to observe maximal PKCμ-S916 or Cx43-S368 phosphorylation. Furthermore, the regulation of Cx43-S368 phosphorylation by PKCμ promotes Cx43 degradation; thus, phosphorylated Cx43 may be internalized and degraded, thereby reducing the observed Cx43-S368 phosphorylation levels between native and wounded tissues. This idea is further supported by the observed perinuclear localization of pCx43-S368 in the cytosol, similar to internalization of Cx43 in HaCaT cells following PMA treatment (Figures 3A and 3B).

In addition, while PMA is known to decrease intercellular communication and induce cell migration and wound healing, our results suggest that the molecular basis of PMA controlling intercellular communication in HaCaT keratinocytes is through the activation of PKCμ-dependent Cx43-S368 phosphorylation to control gap junction function, and the ability of PMA to suppress intercellular communication and promote wound healing is completely blocked by PKCμ inhibition or depletion (Figures 1 and 2). Furthermore, we used Cx43-depletion followed by reconstitution with Cx43 S368 phosphorylation mutants to demonstrate that the phosphorylation of S368 is required for the effects of PKCμ. These findings are further supported by the observation that the ability of PKCμ to regulate intercellular communication and cell migration can be reversed by either the pan-gap junction inhibitor Carbenoxolone or the Cx43-specific inhibitor (and an inducer of S368 phosphorylation) α-CT1, which reduced intercellular communication and accelerated *in vitro* wound healing when cells were treated with PMA and PKCμ was inhibited or depleted. These studies demonstrate a key role for PKCμ in the molecular pathways critical for regulating keratinocyte intercellular communication, cell migration, and *in vitro* wound healing.

PKCμ is known to regulate cell proliferation and de-differentiation in cultured keratinocytes in conditions of low calcium switch.^55^ However, an *in vivo* study demonstrated that PKCμ is dispensable for normal skin homeostasis including epidermal cell proliferation or differentiation.^28^ Upon cellular injury such as wounding, epidermis specific PKCμ knockout mice display delayed wound healing and reduced proliferation in keratinocytes.^28^ Our finding that PKCμ inhibition or depletion delays wound healing *in vitro* is consistent with this *in vivo* study. While other PKC isoforms have been shown to regulate Cx43 phosphorylation at S368, the involvement of PKCμ has not been well characterized, and was considered to have only a minor role.^29^ PKCε and PKCδ phosphorylate Cx43-S368 in osteoblast cell lines and cardiomyocytes, respectively, leading to a reduction in unitary channel conductance.^13^^,^^43^ Until now, PKCμ has not been implicated in regulating Cx43 phosphorylation or function, or intercellular communication. In fact, PKCμ was thought to be of minor consequence to TPA-mediated downregulation of intercellular communication in rat R6 fibroblasts where continued TPA treatment led to a modest downregulation of PKCμ unlike other PKC isozymes such as PKCα, PKCδ, and PKCε.^29^ In contrast to rat fibroblasts, we find that PKCμ plays a key role in maintaining intercellular communication in keratinocytes, suggesting that PKCμ may play a preferential role in regulating Cx43 in a cellular or tissue context-dependent manner, such as in the skin. We further observed that PKCμ can interact with and directly phosphorylate Cx43 at S368. The general consensus recognition sequence of PKCμ is an LxRxxpS/T motif.^56^ For instance, PKCμ phosphorylates Polycystin-2 (TRPP2) at S801 which conforms to the LxRxxpS/T motif.^57^ However, the amino acid sequence RASSR that encompasses Cx43-S368 does not fit to this putative consensus motif. There have been reports of several other PKCμ substrates such as c-Jun and β-catenin that also do not fit this optimal consensus motif.^58^^,^^59^ It has been speculated that these proteins bind to PKCμ at its docking site which facilitates the phosphorylation of sub-optimal motifs present in the substrates.^45^ Therefore, it is plausible to speculate that PKCμ may bind to Cx43 and target S368 for phosphorylation through a similar mechanism.

Our colocalization studies revealed that PKCμ is abundant in the plasma membrane where Cx43 gap junction plaques are present. Prolonged PMA treatment led to dispersal of both PKCμ and Cx43 from the plasma membrane to the cytosol. In many cases, phosphorylation by PKCμ induces interaction of its substrates with the scaffolding proteins such as those within the 14-3-3 family. For instance, 14-3-3 binds to phosphorylated RIN1 and sequesters it to the cytosol, preventing its function at the plasma membrane.^60^^,^^61^ Our results demonstrated that PMA-activated PKCμ colocalizes with Cx43 in the cytosol, reducing the amount of Cx43 present at the plasma membrane. Cycloheximide chase assays further revealed that prolonged PMA treatment reduces total Cx43 levels. However, PKCμ inhibition significantly reduced the rate of Cx43 degradation, suggesting that degradation of Cx43 in response to these stimuli requires PKCμ. Interestingly, 14-3-3 has also been studied in relation to gap junction plaque internalization where 14-3-3τ binding leads to Cx43 ubiquitination, internalization, and degradation.^62^ Therefore, our finding suggests that PKCμ regulates Cx43 in a parallel manner to how PKCμ targets other substrates like RIN1 and given 14-3-3 proteins often bind their targets in a phosphorylation-dependent manner, suggest that PKCμ targeting Cx43 may provide that phospho-binding motif to allow its recognition by 14-3-3. Future studies are warranted to determine if 14-3-3s mediate the internalization of Cx43 following phosphorylation by PKCμ.

Furthermore, identification of PKCμ as a regulator of Cx43 biology suggests that PKCμ may regulate other Cx43 downstream effects, including calcium signaling, cellular electrophysiology, tumorigenesis, and cancer metastasis.^63^^,^^64^^,^^65^ In addition, identifying PKCμ as a key regulator of Cx43-dependent pathways and downstream physiological functions as well as pathophysiology associated with Cx43 dysregulation opens the door to identifying upstream regulators of PKCμ activation and function that may serve as therapeutic targets for wound healing as well as for additional disease settings.

Over the past few decades, gap junction inhibition has been a popular strategy to enhance wound healing,^66^ leading to the assessment of various methods of targeting Cx43 inhibition, such as using antisense oligonucleotides that bind to Cx43 mRNA and connexin mimetic peptides that directly bind to Cx43, such as Gap27.^10^^,^^67^ We utilized two strategies to inhibit gap junctions in the present study. Carbenoxolone is a non-selective inhibitor of gap junctions that shows modest potency, and its therapeutic efficacy has been tested previously in patients with duodenal ulcers.^68^ However, CBX shows an unfavorable safety profile with several side effects, such as hypokalemia, which induces renal and neuromuscular damage with prolonged treatment.^68^ We observed that CBX substantially reduced intercellular communication and increased *in vitro* wound healing in PKCμ inhibited HaCaT cells. The second strategy employed to inhibit gap junction activity was α-CT1, which inhibits the interaction of the Cx43 C-terminal tail with the scaffolding protein ZO-1. Previous studies have found that treatment with α-CT1 also promotes phosphorylation of Cx43-S368 in cardiac ischemia-reperfusion injury models in a PKCε-dependent manner.^69^ Phosphorylation of Cx43-S368 is associated with the reduction of Cx43 channels in the plasma membrane.^13^^,^^14^ Our observation that α-CT1 increases Cx43 phosphorylation at S368 and downregulates Cx43 in scratch wounded HaCaT cells is consistent with these findings. Moreover, reconstitution experiments with Cx43 phosphomimetic substitutions (S368D) suggested that the S368 residue in the Cx43 C-terminal tail is essential for PKCμ-mediated regulation of intercellular communication and wound healing. Furthermore, the degradation of Cx43 in response to PMA appears to be fully dependent on PKCμ. Taken together, these results indicate that PKCμ regulates intercellular communication and cellular migration by controlling gap junction biology.

Interestingly, our data revealed that α-CT1 reduced intercellular communication in HaCaT cells. Animal studies in mice and pigs have shown that α-CT1 increases the wound healing rate.^70^^,^^71^ Clinical trials with α-CT1 have determined that it improves scar visual appearance by 47% in patients who underwent laparoscopic surgery.^72^ Consistent with this finding, we observed that α-CT1 accelerated *in vitro* wound healing in PKCμ-inhibited or depleted HaCaT cells, suggesting that Cx43-S368 phosphorylation may be necessary for PKCμ-mediated control of intercellular communication and wound healing in keratinocytes. Therefore, this study has identified a potential PKCμ pathway that could be included in future studies involving α-CT1 and Cx43 in the context of wound healing.

In conclusion, our study shows that PKCμ controls cell migration in an *in vitro* wound healing model through Cx43-S368 phosphorylation to suppress intercellular communication, providing important insights into the role of PKCμ in skin biology. Cx43 and its controlled downregulation in the wounded epidermis is essential for efficient wound healing, whereas chronic non-healing wounds have been shown to overexpress Cx43.^73^ In addition, the present study suggests that PKCμ may play a key role in regulating Cx43 levels in keratinocytes to further promote wound repair warranting future studies assessing the regulation of Cx43 by PKCμ *in vivo*.

### Limitations of the study

Cx43-S368 is phosphorylated by several other PKC isozymes.^13^^,^^43^ However, it remains unclear how these other PKC isozymes may influence the interaction and targeting of Cx43 by PKCμ. As previous studies have shown that PKCμ itself is a target of DAG activated PKC isozymes, we speculate that PKCμ may act downstream of other PKC isozymes.^74^^,^^75^ This is plausible since a prior study has shown that co-transfection of PKCμ with constitutively active mutants of novel PKCs including PKCs δ, ε, η, and θ leads to activation of PKCμ even in the absence of stimulation.^76^ Similarly, PKC inhibitors that do not directly inhibit PKCμ can effectively inhibit PKCμ activation.^76^

Another limitation of this study is that it does not address other PRKD isozymes, including PRKD2 and PRKD3. All three isoforms are expressed in the skin,^28^ and previous studies have shown that PKCμ reduction alone blocks proliferation and keratinocyte de-differentiation.^55^ However, PRKD2 and PRKD3 could not compensate for the effects of reduced PKCμ in keratinocytes which suggests a major role of PKCμ (PRKD1) in the maintenance of skin biology.^55^ Since our data show that shRNA-mediated PKCμ (PRKD1)-specific knockdown delays *in vitro* wound healing and enhances intercellular communication, other PRKD isozymes may not be important to the proposed signaling pathway. Finally, while epidermis-specific PKCμ knockout mice show defects in wound healing,^28^ the present study is the first to demonstrate that PKCμ may regulate wound healing *in vivo* by controlling Cx43-S368 phosphorylation and gap junction-mediated intercellular communication.

## STAR★Methods

### Key resources table

**Table:** 

REAGENT or RESOURCE	SOURCE	IDENTIFIER
**Antibodies**		

Cx43	Cell Signaling Technology	Cat. #: 3512S; RRID: AB_2294590
pCx43 S368	Cell Signaling Technology	Cat. #: 3511S; RRID: AB_2110169
PKCμ	Cell Signaling Technology	Cat. #: 90039S; RRID: AB_2800149
pPKCμ S916	Cell Signaling Technology	Cat. #: 2051S; RRID: AB_330841
HA	Sigma	Cat. #: H6908; RRID: AB_260070
GST	Santa Cruz Biotechnology	Cat. #: sc-138; RRID: AB_627677
Tubulin	Sigma	Cat. #: T5168; RRID: AB_477579
Alexa Fluor™ 488 donkey anti-rabbit IgG (H+L)	Molecular Probes	Cat. #: A-21206; RRID: AB_2535792
Alexa Fluor™ 568 donkey anti-rabbit IgG (H+L)	Thermo Fisher Scientific	Cat. #: A10042; RRID: AB_2534017
Donkey anti-goat IgG (H+L) Alexa Fluor™ Plus 647	Thermo Fisher Scientific	Cat. #: A32849; RRID: AB_2762840

**Bacterial and virus strains**		

DH5-alpha Competent *E. coli* (High Efficiency)	NEB	Cat. #: C2987
BL21 (DE3) Competent Cells	Thermo Fisher Scientific	Cat. #: ECO114

**Biological samples**		

Human Native Ready-to-use Skin 11 mm Diameter	Genoskin	Batch #: 20230912.2
Human Wound Skin with 2 mm Central Wound	Genoskin	Batch #: 20230912.2

**Chemicals, peptides, and recombinant proteins**		

Calcium Chloride Dihydrate	Acros Organics	Cat. #: 423525000; CAS: 10035-04-8
HEPES	Sigma	Cat. #: H4034; CAS: 7365-45-9
Sodium Chloride	Fisher Bioreagents	Cat. #: BP358-10; CAS: 7647-14-5
Potassium Chloride	Sigma-Aldrich	Cat. #: P9333; CAS: 7447-40-7
Sodium Phosphate Dibasic Anhydrous	Fisher Bioreagents	Cat. #: BP332; CAS: 7558-79-4
Sodium Hydroxide	Fisher Chemical	Cat. #: SS255-1; CAS: 1310-73-2
Sodium Dodecyl Sulfate	VWR Life Science	Cat. #: 0227-1KG; CAS: 151-21-3
Sodium Azide	Sigma-Aldrich	Cat. #: S2002-500G; CAS: 26628-22-8
Sodium Deoxycholate	Sigma-Aldrich	Cat. #: D6750; CAS: 302-95-4
Tween 20	Fisher Bioreagents	Cat. #: BP337-100; CAS: 9005-64-5
Triton X-100	Fisher Bioreagents	Cat. #: BP151-500; CAS: 9002-93-1
Agarose	Thermo Fisher Scientific	Cat. #: R0492; CAS: 9012-36-6
Agar	Fisher Bioreagents	Cat. #: BP9744-500; CAS: 9002-18-0
Bromophenol Blue	Sigma-Aldrich	Cat. #: B0126-25G; CAS: 115-39-9
DMSO	Fisher Bioreagents	Cat. #: BP231-100; CAS: 67-68-5
Glycerol	Fisher Bioreagents	Cat. #: BP229-1; CAS: 56-81-5
Ethidium Bromide Solution	Invitrogen	Cat. #: 15585011; CAS: 1239-45-8
EDTA	Fisher Bioreagents	Cat. #: BP118-500; CAS: 60-00-4
Glycine	Thermo Fisher Scientific	Cat. #: A13816.0C; CAS: 56-40-6
LB Broth	Fisher Bioreagents	Cat. #: BP9731-5; CAS: 56-40-6
Tris Base	EMD Millipore Corp	Cat. #: 648310-2.5KG; CAS: 77-86-1
2-Propanol	Fisher Bioreagents	Cat. #: A416P-4; CAS: 67-63-0
Acetic Acid	Fisher Bioreagents	Cat. #: A38-212; CAS: 64-19-7
Hydrochloric Acid	Fisher Bioreagents	Cat. #: A481-212 CAS: 7647-01-0
Ethanol	Decon Labs, Inc.	Cat. #: UN1170; CAS: 64-17-5
Methanol	EMD Millipore Corp	Cat. #: MX0490-4; CAS: 67-56-1
Cycloheximide	Sigma	Cat. #: 01810-5G; CAS: 66-81-9
Ampicillin Sodium Salt	Fisher Bioreagents	Cat. #: BP1760-25; CAS: 69-52-3
Glutathione (Reduced)	Fisher Bioreagents	Cat. #: BP2521-5; CAS: 70-18-8
IPTG	Sigma	Cat. #: I6758-1G; CAS: 367-93-1
Phenylmethanesulfonyl Fluoride	Sigma-Aldrich	Cat. #: P7626-5G; CAS: 329-98-6
Fluoroshied with DAPI	Sigma-Aldrich	Cat. #: F6057-20ml
Carbenoxolone Disodium Salt	Thermo Fisher Scientific	Cat. #: J63714.03; CAS: 7421-40-1
Bovine Serum Albumin	Fisher Bioreagents	Cat. #: BP1605-100; CAS: 9048-46-8
Paraformaldehyde Solution	ChemCruz	Cat. #: sc-281692; CAS: 30525-89-4
Dithiothreitol (DTT)	Fisher Bioreagents	Cat. #: BP172-25; CAS: 3483-12-3
30% Acrylamide/Bis Solution 37.5:1	Bio-Rad	Cat. #: 1610158
Puromycin	Thermo Fisher Scientific	Cat. #: J67236.XF; CAS: 53-79-2
Polybrene	Santa Cruz	Cat. #: sc-134220; CAS: 28728-55-4
N, N, N’, N’-Tetramethyl Ethylenediamine	Acros Organics	Cat. #: 433831000; CAS: 110-18-9
Halt Protease and Phosphatase Inhibitor Cocktail	Thermo Fisher Scientific	Cat. #: 78442
Lucifer Yellow CH	Invitrogen	Cat. #: L453
Magnesium Chloride Hexahydrate	Sigma-Aldrich	Cat. #: M9272-500G; CAS: 7791-18-6
Kinase buffer	Cell Signaling Technology	Cat. #: 9802S
Lysozyme	Sigma	Cat. #: L6876-1G; CAS: 12650-88-3
ATP	Cell Signaling Technology	Cat. #: 9804S; CAS: 987-65-5
Protein A/G Plus	Santa Cruz	Cat. #: SC-2003
Brilliant Blue G-250	Fisher Scientific	Cat. #: BP100-25; CAS: 6104-58-1
Ponceau S	Sigma-Aldrich	Cat. #: P3504-100G; CAS: 6226-79-5
Pierce Anti-HA Agarose	Thermo Fisher Scientific	Cat. #: 26181
Collagen Type I, Rat Tail	EMD Millipore Corp	Cat. #: 08-115
Phorbol 12-myristate 13-acetate (PMA)	Thermo Fisher Scientific	Cat. #: J63916.MCR CAS: 16561-29-8
CRT 0066101	Tocris	Cat. #: 4975; CAS: 1883545-60-5
Lambda Protein Phosphatase	New England Biolabs	Cat. #: P0753S
Trypan Blue	Invitrogen	Cat. #: T10282; CAS:72-57-1
Mitomycin C	Roche	Cat. #: 1010740900; CAS: 50-07-7
Antennapedia (H-RQPKIWFPNRRKPWKK-OH)	Biosynth	Lot #: LP11809
α-CT1 RIS (H-RQPKIWFPNRRKPWKKIELDDPRPR-OH)	Biosynth	Lot #: LP11810
α-CT1 (H-RQPKIWFPNRRKPWKKRPRPDDLEI-OH)	Biosynth	Lot #: BU19813
HA-PKCμ	This manuscript	N/A
HA-PKCμ (K612W)	This manuscript	N/A
GST-Cx43	This manuscript	N/A
GST	This manuscript	N/A

**Experimental models: Cell lines**		

HEK293T	ATCC	Cat. #: CRL-3216; RRID: CVCL__0063
HaCaT	Norbert E. Fusenig, German Cancer Research Center (DKFZ), Heidelberg, Germany.	N/A

**Oligonucleotides**		

shRNA PKCμ A	CCCACGCTCTCTTTGTTCATT	This manuscript
shRNA PKCμ B	CAGGAAGAGATGTAGCTATTA	This manuscript
Cx43 shRNA	GGTGGTAATTGTGGCTAAATA	This manuscript

**Recombinant DNA**		

pGEX-4T-3	Amersham	Available from other sources
GST-Cx43	This manuscript	N/A
HA-PKCμ	Addgene	Plasmid #: 10808
HA-PKCμ (K612W)	Addgene	Plasmid #: 10809
pcDNA3.1 (+)	Thermo Fisher Scientific	Cat. #: V79020
pLKO.1 shRNA EGFP	Addgene	Plasmid #: 30323
pLKO.1 shRNA PKCμ A	This manuscript	N/A
pLKO.1 shRNA PKCμ B	This manuscript	N/A
psPAX2	Addgene	Plasmid #: 12260
pMD2.G-VSV.G	Addgene	Plasmid #: 12259
pLKO.1 shRNA Cx43	This manuscript	N/A
pLenti-GFP-Blasticidin	This manuscript	N/A
pLenti-Cx43-Blasticidin	This manuscript	N/A
pLenti-Cx43 (S368D)-Blasticidin	This manuscript	N/A
pLenti-Cx43 (S368A)-Blasticidin	This manuscript	N/A

**Critical commercial assays**		

Immobilon Western Chemiluminescent HRP Substrate	Millipore	Cat. #: WBKLS0500
DC Protein Assay	Bio-Rad	Cat. #: 5000114
Pierce Glutathione Superflow Agarose	Thermo Fisher Scientific	Cat. #: 25236
PCR and Gel Cleanup Kit	Qiagen	Cat. #: 28506
T4 DNA Ligase	New England Biolabs	Cat. #: M0202
Amicon Ultra-15 Centrifugal Filters -100K	Merck Millipore	Cat. #: UFC910096
Sonic Dismembrator	Fisher Scientific	Cat. #: FB-705
Nitrocellulose Membranes, 0.45μM	Bio-Rad	Cat. #: 162-0251
Bright-line Hemacytometer	Hausser Scientific	Cat. #: Z359629-1EA

**Software and algorithms**		

Image Lab™ Software (Version 5.2.1)	Bio-Rad	RRID:SCR_014210
Illustrator 2023	Adobe	N/A
Prism 9 Version 9.5.0	GraphPad Software	RRID:SCR_002798
ImageJ	National Institutes of Health	RRID:SCR_003070
Gen5 3.10	BioTek	RRID:SCR_017317
NIS-Elements AR 5.02.091 64-bit	Nikon	RRID:SCR_014329

**Other**		

Forma Steri-Cycle CO_2_ Incubator	Thermo Fisher Scientific	Cat. #: 381
IsoTemp 220	Fisher Scientific	Cat. #: 15-462-20Q
Micro12	EKF Diagnostics	Cat. #: 23-550-103
Mighty Slim Power supply	Hoeter	Cat. #: SX259
PowerPac	Bio-Rad	Cat. #: 1645050
Mini-PROTEAN Glass Plates	Bio-Rad	Cat. #: 1653311
Mini Trans-Blot Foam Pads	Bio-Rad	Cat. #: 1703933
Molecular Imager ChemiDoc XRS+	Bio-Rad	Cat. #: 1708265
BioTek Cytation 5 Imaging Reader	Agilent	N/A
C25 Incubator Shaker	New Brunswick Scientific	Cat. #: M1246-0000
Avanti J-E Centrifuge	Beckman Coulter	Cat. #: 369001

### Resource availability

#### Lead contact

Further information and requests for resources and reagents should be directed to and will be fulfilled by the lead contact, Brian J. North (BrianNorth@creighton.edu).

#### Materials availability

Materials established in this study are available from the lead contact upon request.

#### Data and code availability

• All data reported in this paper will be shared by the lead contact upon request.
• This paper does not report original code.
• Any additional information required to reanalyze the data reported in this paper is available from the lead contact upon request.

### Experimental model and study participant details

HEK 293T cells were obtained from the American Type Culture Collection (ATCC). HaCaT cells were previously characterized.^77^ Cells were cultured in Dulbecco's modified Eagles' medium (DMEM) supplemented with 10% Fetal Bovine Serum (FBS), 100 units/ml penicillin, and 100 μg/ml streptomycin (GIBCO) and incubated at 37°C in a humidified atmosphere with 5% CO_2_. Wounded and unwounded human *ex vivo* skin samples were purchased from Genoskin in compliance with IRB approval, federal law, and HIPAA guidelines. Tissues were obtained from the abdomen of a 56 year old African American female.

### Method details

#### Cell treatments and transfections

Treatments including PMA, CRT 0066101, Carbenoxolone, peptides, and cycloheximide were added to the cell culture media at concentrations and times as indicated in figure legends. DMSO was added as vehicle control at the same percentage as treatment group.

For transient transfections of the control vector, HA-PKCμ, or HA-PKCμ (K612W), HEK 293T cells were seeded in 10 cm plates and grown to 80% confluence before being transfected with 5 μg plasmid DNA using the calcium phosphate transfection method. Cells were harvested 48 hours post transfection. To produce viral shRNA containing viral particles, HEK 293T cells were seeded as described above and transfected with 5 μg of the pLKO.1 lentiviral transfer vector, 3.5 μg of the packaging vector (psPAX2), and 1.5 μg of the envelope vector (pMD2.G) using the calcium phosphate transfection method.

#### Cloning

Oligonucleotides utilized for PKCμ shRNA plasmid production are listed under key resources. shRNA plasmids were generated as described in pLKO.1-TRC cloning protocol version 1.0 (Addgene). Briefly, oligonucleotides were annealed in the presence of 10X NEB buffer 2 at 95**°**C for 4 minutes and allowed to cool slowly overnight. The pLKO.1 TRC cloning vector was digested with AgeI and EcoRI in 10X NEB buffer 1. Digested fragments were run on an agarose gel, and DNA was purified using a Qiagen Gel Extraction Kit, following the manufacturer’s protocol. The digested pLKO.1 vector was ligated to the annealed oligos with T4 DNA ligase and transformed into bacteria. DNA was extracted from bacterial colonies and sequenced to confirm insertion of proper shRNA fragment.

To produce GST tagged Cx43, full-length Cx43 plasmid was purchased from Dharmacon. Both pGEX-4T and Cx43 vectors were digested with BamHI and purified using a Qiagen Gel Extraction Kit. Ligations, DNA purifications, and sequencing were performed as described above.

To generate expression vectors containing wild-type, S368D, and S368A amino acid substitutions in Cx43, Cx43 cDNA was subcloned into the NotI sites of pcDNA3.1 (+) (Invitrogen) using standard PCR-based strategies and confirmed by sequencing. The resulting plasmid served as the template for generating amino acid substitutions using the QuikChange Site-Directed Mutagenesis strategy, and the resulting clones were sequenced to confirm the expected base pair substitutions. These mutant constructs were then subcloned into the pLenti-CMV-EGFP-Blast vector, which was linearized by EcoRI and BamHI, followed by insertion of Cx43 using standard PCR-based strategies. To generate pLenti-CMV-EGFP-Blast, the SV40-puro coding sequence was removed from pLenti-CMV-MCS-GFP-SV-Puro (a gift from Paul Odgren, Addgene plasmid #73582; http://n2t.net/addgene:73582;RRID: Addgene_73582)^78^ by MluI/EcoRI digest and replaced with a PCR amplified WPRE cassette at this site using In-Fusion cloning. PCR-amplified hPGK-BSD was subsequently inserted into the BamHI site between EGFP and WPRE elements to produce the final vector, which was confirmed by whole plasmid sequencing.

#### Connexin 43 reconstitution

Viral particles containing shRNA targeting Cx43 was generated as described above and used to infect HaCaT cells, followed by Puromycin (10 μg/ml) selection. Whole cell extracts were prepared and used for western blotting to determine the successful depletion of Cx43 in HaCaT cells, using pLKO.1 expressing shGFP as a control. To reconstitute Cx43-depleted cells with wild-type (WT), S368D, or S368A, viral particles were generated using the pLenti-CMV-Blasticidin containing each version of Cx43 as well as an empty vector, as described above, and subsequently used to infect the Cx43-depleted HaCaT cells followed by selection with Blasticidin (10 μg/ml).

#### Western blotting

Cells were lysed in IPLS buffer (50 mM Tris-HCl, 0.5 mM EDTA, 150 mM NaCl, 0.5% NP-40, 1X HALT protease and phosphatase inhibitor cocktail) for 30 minutes on ice with agitation followed by centrifugation at 13,000 rpm at 4**°**C for 5 min. The protein concentration was quantified with DC Protein Assay Kit. Protein concentrations were normalized and Laemmli buffer was added to 1X concentration. Proteins were boiled for 10 minutes at 95**°**C before separation by SDS-PAGE on a 10% gel. The resolved gels were transferred to a nitrocellulose membrane, and the membranes were blocked with 5% dry milk diluted in 1xTBS with 0.1% Tween-20, followed by incubation with primary antibody overnight at 4**°**C, washed, and subsequently incubated with anti-rabbit or anti-mouse HRP conjugated secondary antibodies for 45 minutes at room temperature. Membranes were washed extensively followed by addition of ECL substrate according to the manufacturer’s recommendations and detection on Bio-Rad ChemiDoc XRS+ molecular imager.

#### Lentiviral infection

Virus containing media was harvested at 48 hours post transfection and filtered through 0.45 μM PES membrane. HaCaT cells were transduced with virus containing media (diluted 1:1 with fresh media) in the presence of 10 μg/ml polybrene. Puromycin selection was initiated 48 hours after infection and terminated once uninfected control cells died. Western blot analysis and qPCR were utilized to confirm PKCμ knockdown.

#### Scrape loading/dye transfer assay (SL/DT assay)

SL/DT assay was performed as previously described.^40^ Briefly, HaCaT cells were seeded in a six well plate and grown to 100% confluency. Media was removed and cells were washed twice with PBS. Lucifer yellow dye (1 mg/ml) was dissolved in PBS and added to the cell monolayer. A feathered scalpel blade was rolled across the monolayer to create a single cut. Cells were incubated in the dye mixture for 10 minutes at 37**°**C. Following incubation, cells were washed twice with PBS and then fixed with 4% paraformaldehyde. Images of the cut were taken with a Nikon eclipse Ti2, and the dye migration area was quantified with NIS-elements AR analysis software.

#### *In vitro* kinase assay

HEK 293T cells were transfected with control vector and HA-PKCμ plasmids as described above. Cells were lysed in IPLS buffer for 10 minutes at 4**°**C and subsequently sonicated with a microtip at 50% amplitude for two pulses at 10 seconds each. Cell lysates were centrifuged for 20 minutes at 13,000 rpm at 4**°**C. The supernatants were transferred to new tubes and HA beads were added to the lysates and incubated for 3 hours at 4**°**C with constant agitation. Immunoprecipitates were pelleted by centrifugation at 3,000 rpm for 1 minute. The lysate was aspirated, and the remaining HA beads were washed three times in PBS buffer. To prepare GST tagged recombinant Cx43, BL21 *E. coli* was transformed with pGEX-4T-3 (GST) and pGEX-4T-3-Cx43 (GST-Cx43) vectors. A single colony was picked and grown in 1L of Luria Broth until the OD reached 0.4. Protein expression was induced with 0.5 μM IPTG and incubated at 37**°**C for 3 hours. Bacteria were pelleted at 6,000 rpm for 15 minutes at 4**°**C in a Beckman Coulter centrifuge with JLA 16.250 rotor. The bacterial pellet was washed with PBS, resuspended in PBS, and sonicated for 10 times for 30 seconds at 50% amplitude. Bacterial debris was pelleted at 12,000 rpm for 12 min at 4**°**C in a Beckman Coulter centrifuge with JLA 16.250 rotor. Supernatant was transferred to a new tube and Glutathione Superflow Agarose was added and incubated for 2 hours at 4**°**C. GST and GST-Cx43 were then isolated according to the manufacturer’s protocol.

*In vitro* kinase assay was carried out with 4 μg GST or GST-Cx43 and agarose-conjugated HA-PKCμ protein isolated from HEK 293T cells combined with kinase buffer from Cell Signaling Technology in the absence or presence of ATP and incubated for 30 minutes at 30**°**C. Reactions were terminated by addition of 1X Laemmli buffer. Samples were prepared and subjected to SDS-PAGE followed by western blotting as described above.

#### *In vitro* pull-down assay

HA-PKCμ, GST, and GST-Cx43 were prepared as previously described. GST-CX43 or GST proteins diluted in buffer (125 mM Tri-HCl, pH 8.0, 150 mM NaCl) were added to the HA beads and incubated for 3 hours at 4**°**C. The beads were washed three times with cold PBS and resuspended in 60 μl 1X Laemmli buffer. Samples were prepared and subjected to SDS-PAGE followed by western blotting as described above.

#### Cycloheximide chase assay

Cycloheximide chase assay was conducted on 100% confluent HaCaT cells treated with cycloheximide (100 μg/ml) in the presence or absence of PMA and/or CRT at indicated time points. Cells were washed with cold PBS and lysed with IPLS buffer for 30 minutes with constant agitation. Cellular debris was pelleted at 13,000 rpm for 5 min at 4**°**C. Lysates were then treated with Lambda protein phosphatase at 30**°**C for 30 minutes according to the manufacturer’s instructions to dephosphorylate Cx43. Total protein was normalized using DC Protein Assay Kit. Samples were diluted in 1X Laemmli buffer and subjected to SDS-PAGE followed by western blotting as described above.

#### Immunofluorescence

Chamber slides (ibidi) were treated with 80 μg/ml rat collagen for 1 hour at 37°C/5% CO_2_ incubator and washed quickly with PBS. HaCaT cells (1x10^4^ cells) were seeded in 8-well chamber slides and grown for 24 hours. Cells were treated with either DMSO or PMA for 3 hours, rinsed with PBS, and fixed with 4% Paraformaldehyde (PFA) for 15 minutes at room temperature followed by three washes with PBS. Cells were permeabilized with 0.5% Triton-X-100 for 30 minutes at room temperature and washed as described above. Cells were blocked with 0.1% BSA in PBS at room temperature for 60 minutes and subsequently incubated with primary antibodies diluted 1:100 in 0.1% BSA and incubated overnight at 4°C. Cells were rinsed three times with PBS and incubated with Alexa fluor-conjugated secondary antibodies at room temperature for 30 minutes followed by washing three times with PBS. Slides were then mounted with mounting medium with DAPI. Chamber slides were scanned with Olympus VSI slide scanner. To assess colocalization across a linear region of interest, a line was applied to each image and signal intensity was measured using ImageJ software and plotted. To determine staining intensity of pPKCμ-S916 in cells adjacent to the scratch wound compared to cells internal to the scratch wound, 10 random fields adjacent to the scratch and 10 random fields internal to the scratch were measured using ImageJ, values were averaged, and the ratio of signal intensity from adjacent and internal fields was calculated. Calculations from three independent images were averaged and a t-test was carried out for statistical analysis between indicated time points.

For time-dependent colocalization analysis, HaCaT cells were grown on chamber slides to 100% confluency. Chambers were treated with either DMSO or PMA for 0, 30, 60, or 120 minutes. Immunofluorescence was carried out as described above. Images were acquired using a Nikon Ti-E inverted microscope with a Yokagawa Spinning Disc and a Plan Apo λ 100x Oil objective. Nikon NIS software was used to perform 3D Richardson-Lucy deconvolution for each image.

#### Immunofluorescence in skin biopsies

Skin biopsies were maintained in culture medium provided by Genoskin for 2 hours at 37°C in a humidified atmosphere with 5% CO_2_. The skin biopsies samples were washed in PBS and fixed in formalin overnight at room temperature, followed by a 70% ethanol wash. The skin biopsies were then processed to obtain Formalin fixed paraffin embedded (FFPE) sections. Briefly, both wounded and unwounded samples were washed twice in xylene for 5 minutes to remove the paraffin. Tissue samples were rehydrated in 100%, 95%, 75%, and 50% ethanol solutions for 5 minutes each followed by heat activated antigen retrieval. Sodium citrate buffer (10 mM sodium citrate, 0.05% Tween 20, pH 6.0) was used for antigen retrieval, which was boiled in a microwave for 6 minutes and transferred to a steamer in which the tissue slides were placed for 30 minutes followed by a 20-minute cool down process. Tissue slides were then incubated in blocking buffer (3% BSA in PBS) for 30 minutes at room temperature. Primary antibody (diluted 1:500 in 1% BSA/PBS) were added to each tissue section and incubated at 4**°**C overnight. Tissue slides were washed with TBS-T three times and secondary antibody (diluted 1:1000 in 1% BSA/PBS) was added to each tissue section and incubated for 1 hour at room temperature in a dark humidifying chamber. The slides were washed three times with TBS-T and mounted with DAPI containing Fluoroshield mounting medium. Confocal images were acquired as described above. To determine the staining intensity of pPKCμ-S916 and pCx43-S368, 10 random fields were measured using ImageJ, the values were averaged, and the ratio of signal intensity from pPKCμ-S916 was normalized to total PKCμ, and signal intensity from pCx43-S368 was normalized to total Cx43. Comparisons between unwounded and wounded tissues were conducted and t-tests were carried out for statistical analysis between unwounded and wounded tissues.

#### Quantitative *In vitro* scratch assay

*In vitro* wound healing assays were carried out as previously described.^79^ Briefly, HaCaT cells were seeded in 6-well plates and grown to 100% confluency before being treated with 10 μg/mL mitomycin C for 2 hours. Cells were washed twice with PBS after which a scratch across the monolayer was made with a 200 μL pipette tip. DMEM was added to the cells along with the indicated treatments. Images were taken at 0 hour and 16 hours with a Nikon eclipse Ti2 and quantified by NIS-elements AR analysis software.

### Quantification and statistical analysis

All results were analyzed by GraphPad Prism version 9.0 (GraphPad Software). The data were expressed as mean ± SD. All comparisons between two groups were assessed by Student’s t tests. p < 0.05 was defined as statistical significance where ^∗^p < 0.05, ^∗∗^p < 0.01, ^∗∗∗^p < 0.001.
