# Supplementary material for: PKCμ promotes keratinocyte cell migration through Cx43 phosphorylation-mediated suppression of intercellular communication
Source: iScience. 2024 Jan 29;27(3):109033. doi: 10.1016/j.isci.2024.109033 (PMC10875573; doi:10.1016/j.isci.2024.109033)
Supplement: Document S1. Figures S1–S7 [file mmc1.pdf]

iScience, Volume *27*

Supplemental information

PKCμ promotes keratinocyte cell migration through Cx43 phosphorylation-mediated suppression of intercellular communication

Renju Pun, Ann M. Cavanaugh, Emily Aldrich, Olivia Tran, Justin C. Rudd, Laura A. Hansen, and Brian J. North

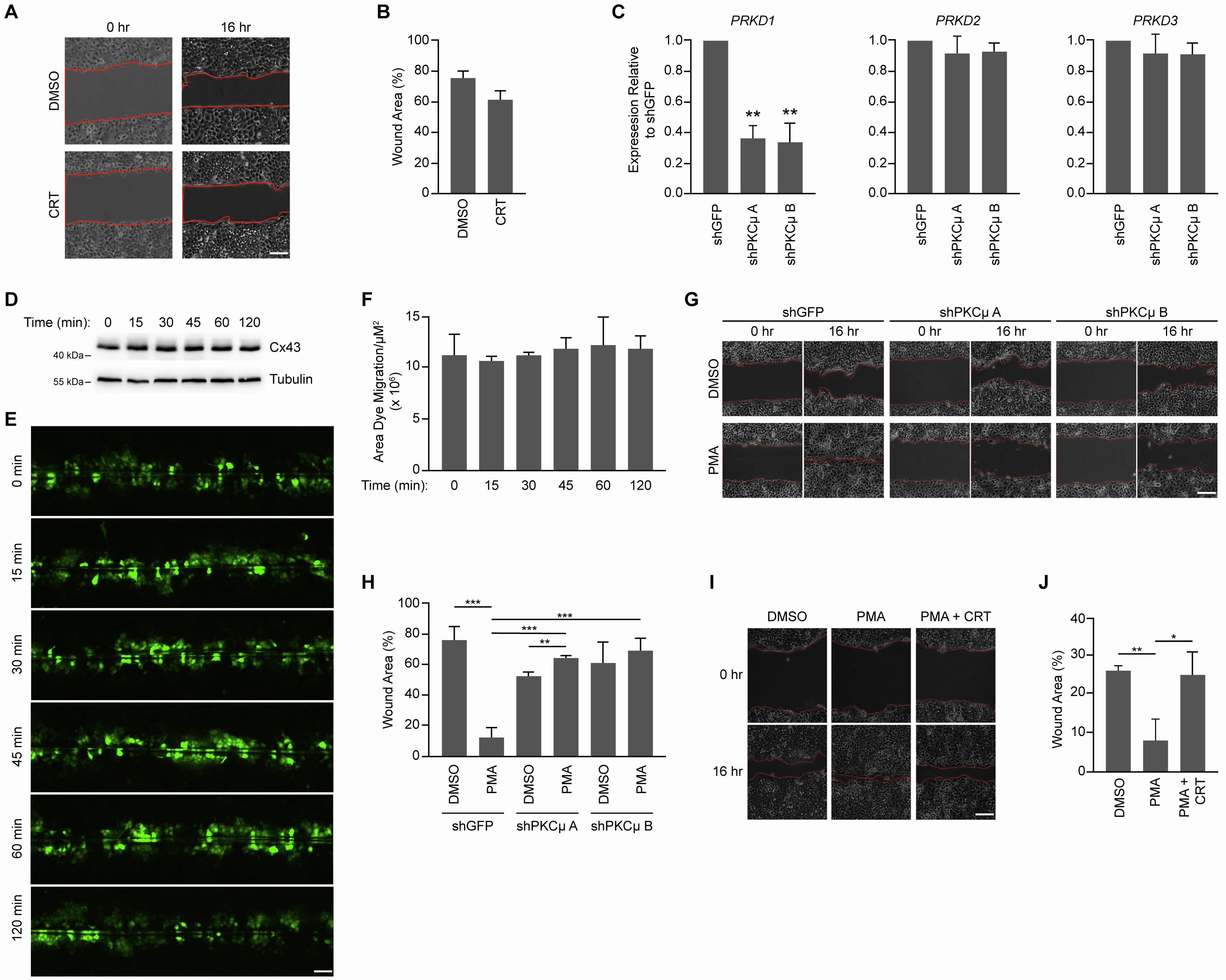
A
0 hr
16 hr
DMSO
CRT
B
Wound Area (%)
DMSO
CRT
C
PRKD1
PRKD2
PRKD3
Expresesion Relative to shGFP
shGFP
shPKCμ A
shPKCμ B
D
Time (min): 0 15 30 45 60 120
Cx43
Tubulin
40 kDa
55 kDa
E
0 min
15 min
30 min
45 min
60 min
120 min
F
Area Dye Migration/μM² (x 10⁶)
Time (min): 0 15 30 45 60 120
G
shGFP
shPKCμ A
shPKCμ B
0 hr
16 hr
DMSO
PMA
H
Wound Area (%)
DMSO
PMA
shGFP
shPKCμ A
shPKCμ B
I
DMSO
PMA
PMA + CRT
0 hr
16 hr
J
Wound Area (%)
DMSO
PMA
PMA + CRT

Figure S1: PKCμ inhibition with CRT does not influence *in vitro* wound healing, related to Figure 1. (A) Representative images of *in vitro* wound healing assay of HaCaT cells treated with CRT (1 μM) at 0 and 16 hours. Scale bar = 100 μm. (B) Percentage of wound area remaining after 16 hours from three replicate experiments as shown in (A). (C) qPCR analysis of *PRKD1*, *PRKD2*, and *PRKD3* mRNA levels in HaCaT cells treated with shGFP, shPKCμ A, or shPKCμ B. (D) Western blot showing total Cx43 post following treatment with mitomycin C (5 μM). (E) Representative fluorescent microscopic images of SL/DT assay in HaCaT cells treated with mitomycin C for indicated time points. Scale bar = 100 μm. (F) Quantification of area of dye migration based on three replicates of experiment shown in (D). (G) Representative images of *in vitro* wound healing assay of serum starved HaCaT cells expressing shGFP, shPKCμ A, or shPKCμ B at 0 and 16 hours. Scale bar = 100 μm. (H) Percentage of remaining wound area at 16 hours of experiment shown in (G). (I) Representative images of *in vitro* wound healing assay of HaCaT cells under normal growth conditions at 0 and 16 hours. Scale bar = 100 μm. (J) Percentage of remaining wound area at 16 hours of experiment shown in (I). All calculations are based on three replicates -/+ S.D. *p<0.05, **p<0.01, ***p<0.001 (Student's t-test).

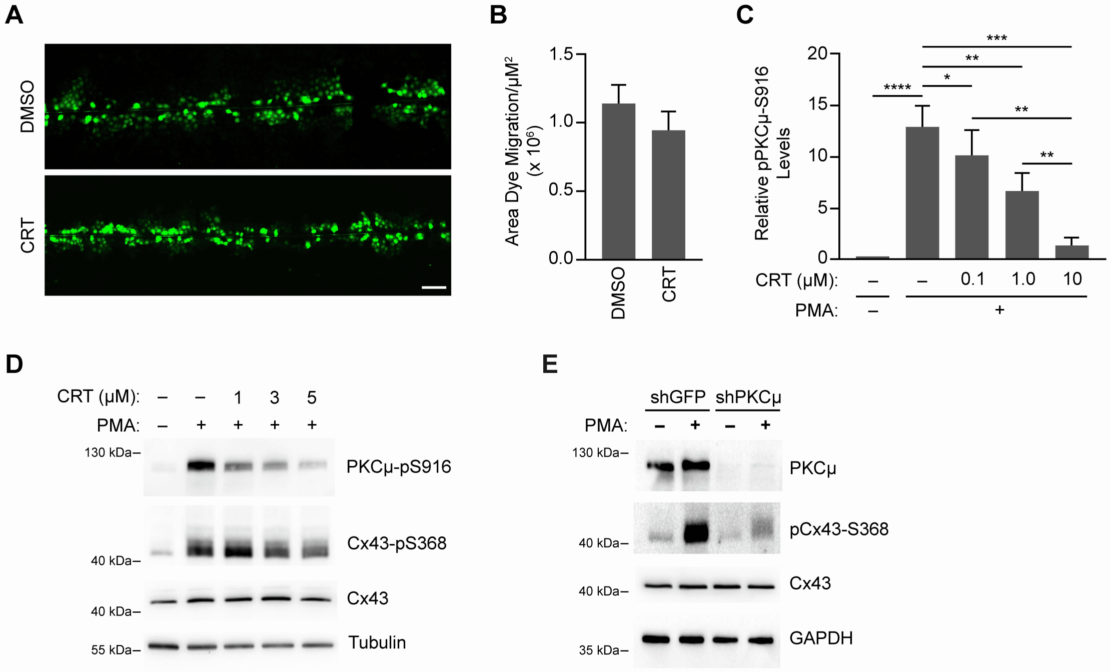

A
DMSO
CRT
B
Area Dye Migration/μM²
(x 10⁶)
1.5
1.0
0.5
0.0
DMSO
CRT
C
Relative pPKCμ-S916 Levels
20
15
10
5
0
****
*
**
***
**
**
CRT (μM):
–
–
0.1
1.0
10
PMA:
–
+
D
CRT (μM):
–
–
1
3
5
PMA:
–
+
+
+
+
130 kDa–
PKCμ-pS916
Cx43-pS368
40 kDa–
Cx43
40 kDa–
Tubulin
55 kDa–
E
shGFP
shPKCμ
PMA:
–
+
–
+
130 kDa–
PKCμ
pCx43-S368
40 kDa–
Cx43
40 kDa–
GAPDH
35 kDa–

Figure S2: PKCμ inhibition with CRT alone does not affect dye migration through gap junctions, related to Figure 2. (A) Representative fluorescent microscopic images of SL/DT assay in HaCaT cells treated with CRT (5 μM) for 6 hours. Scale bar = 100 μm. (B) Quantification of dye migration area from three replicate experiments as shown in (A). (C) Quantification of pPKCμ-S916 following treatment with CRT at indicated concentrations compared to DMSO control based on densitometric analysis from three replicate experiments as shown in (Figure 2F). (D) MEF cells treated with an increasing concentration of CRT in the presence or absence of 0.5 μM PMA and probed for pPKCμ-S916, pCx43-S368, total Cx43, and Tubulin. (E) MEF cells expressing shGFP (control) and shPKCμ treated with or without 0.5 μM PMA and probed for total PKCμ, pCx43-S368, total Cx43, and GAPDH. All calculations are based on three replicates -/+ S.D. *$p<0.05$, **$p<0.01$, ***$p<0.001$ (Student's t-test).

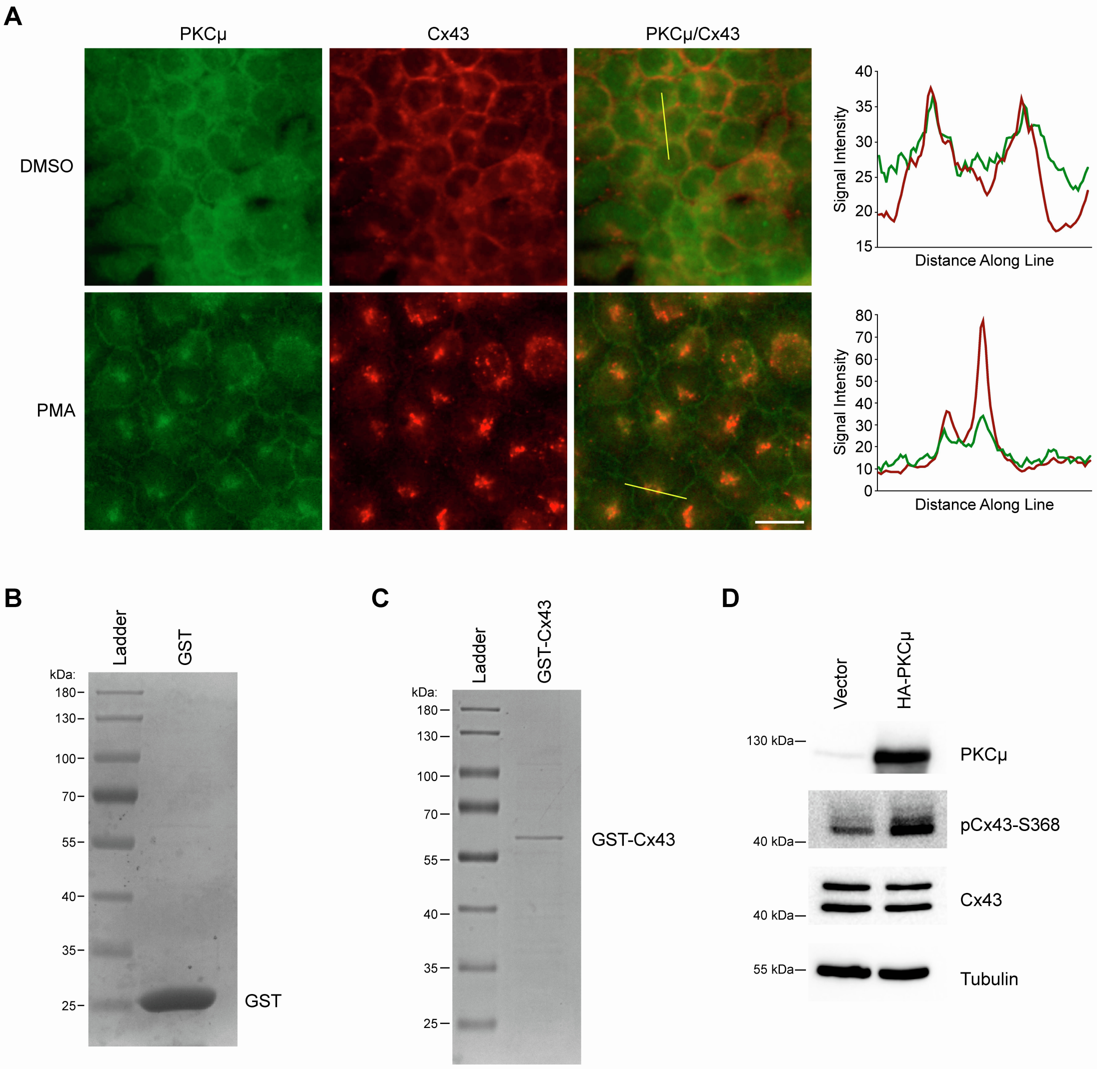
A
PKCμ
Cx43
PKCμ/Cx43
DMSO
PMA
Signal Intensity
Distance Along Line
B
Ladder
GST
kDa:
180
130
100
70
55
40
35
25
GST
C
Ladder
GST-Cx43
GST-Cx43
D
Vector
HA-PKCμ
130 kDa
PKCμ
40 kDa
pCx43-S368
40 kDa
Cx43
55 kDa
Tubulin

Figure S3: PKCμ and Cx43 colocalize at the cell membrane and in the cytosol post PMA treatment, related to Figure 3. (A) Representative immunofluorescence images of HaCaT cells treated with or without PMA (0.5 μM) stained for PKCμ and Cx43. Signal intensity plots of both channels across indicated line in merged image is shown. Scale bar = 20 μm. (B) Coomassie blue stained SDS-PAGE gel of purified recombinant GST protein (26 kDa) from BL21 *E. coli*. (C) Coomassie blue stained SDS-PAGE gel of purified recombinant GST-Cx43 protein (69 kDa) from BL21 *E. coli*. (D) Western blots of cell lysates from 293T cells transfected with control vector or HA-PKCμ probed pPKCμ, pCx43-S368, total Cx43, and Tubulin.

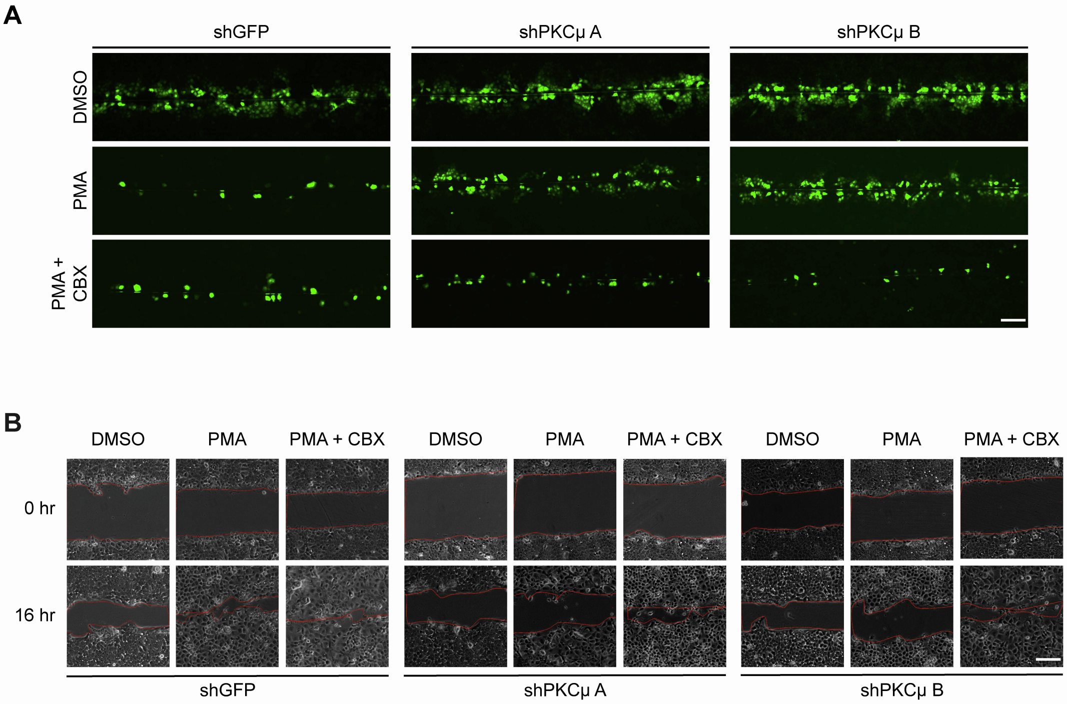
A
shGFP
shPKCμ A
shPKCμ B
DMSO
PMA
PMA + CBX
B
DMSO
PMA
PMA + CBX
DMSO
PMA
PMA + CBX
DMSO
PMA
PMA + CBX
0 hr
16 hr
shGFP
shPKCμ A
shPKCμ B

Figure S4: Inhibition of Cx43 with CBX reduces intercellular communication through gap junctions, related to Figure 5. (A) Representative fluorescence microscopic images of SL/DT assay in HaCaT cells expressing shGFP, shPKCμ A, or shPKCμ B, and in the absence or presence of CBX (100 μM) for 6 hours, followed by PMA (0.5 μM) for 30 min. Scale bar = 100 μm. (B) Representative images of *in vitro* wound healing assay of HaCaT cells expressing shGFP, shPKCμ A, or shPKCμ B treated with PMA (10 nM) with or without CBX (50 μM) at 0 and 16 hours. Scale bar = 100 μm.

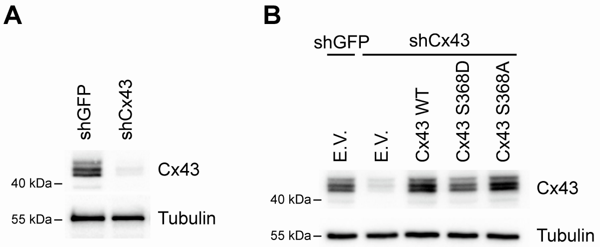

A
shGFP
shCx43
Cx43
40 kDa
55 kDa
Tubulin
B
shGFP
shCx43
E.V.
E.V.
Cx43 WT
Cx43 S368D
Cx43 S368A
Cx43
40 kDa
55 kDa
Tubulin

Figure S5: Cx43-depletion and reconstitution with Cx43 wild-type, S368D, and S368A in HaCaT cells, related to Figures 5 and 6. (A) Western blot of cell lysates from HaCaT cells expressing shGFP and shCx43 and probed for Cx43 and Tubulin. (B) Western blot of cell lysates from shCx43 expressing HaCaT cells reconstituted with ectopic expression of Cx43 wild-type, S368D, and S368A probed for Cx43 and Tubulin.

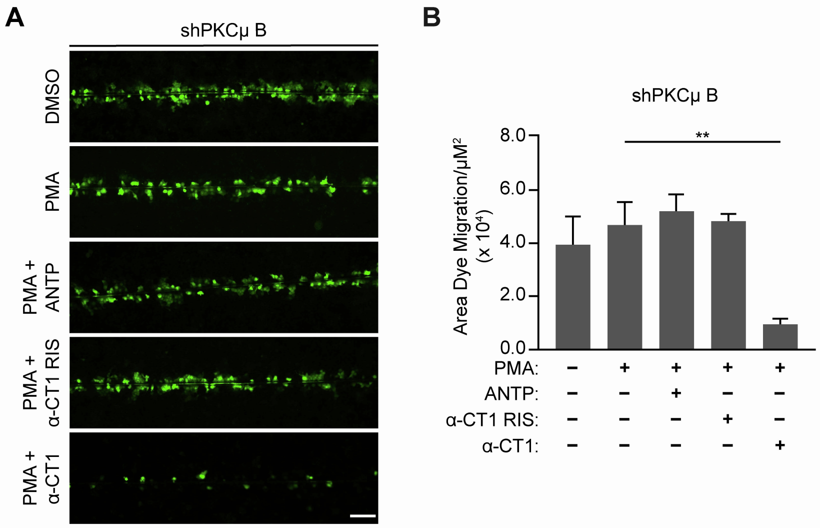
A
shPKCμ B
DMSO
PMA
PMA + ANTP
PMA + α-CT1 RIS
PMA + α-CT1
B
shPKCμ B
Area Dye Migration/μM²
(x 10⁴)
8.0
6.0
4.0
2.0
0.0
**
PMA: − + + + +
ANTP: − − + − −
α-CT1 RIS: − − − + −
α-CT1: − − − − +

Figure S6: Inhibition of Cx43 with α-CT1 reduces intercellular communication through gap junctions, related to Figure 7. (A) Representative fluorescent microscopic images of SL/DT assay in HaCaT cells expressing shPKCμ B, and in the absence or presence of ANTP, α-CT1 RIS, or α-CT1 peptides (each at 100 μM) for 6 hours followed by PMA (10 nM) for 30 min. Scale bar = 100 μm. (B) Quantification of dye migration area from three replicate experiments as shown in (A). All calculations are based on three replicates -/+ S.D. ***p*<0.01 (Student's t-test).

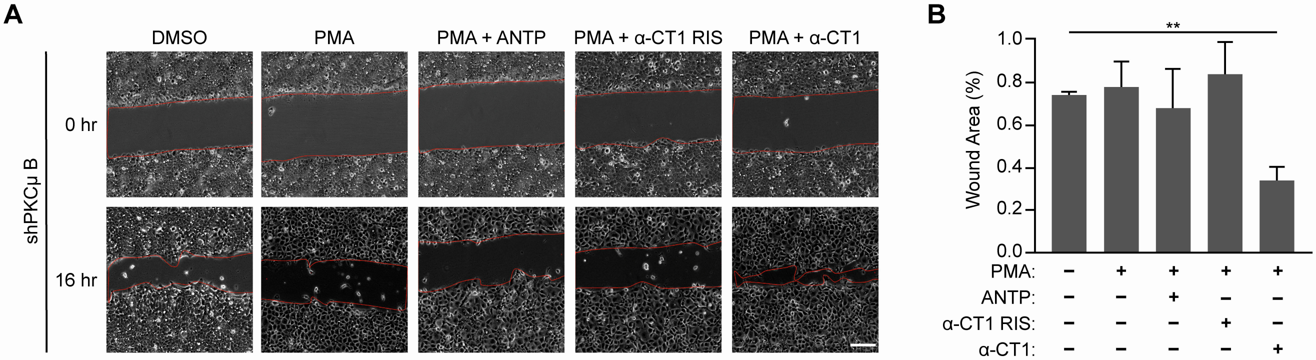
A
DMSO
PMA
PMA + ANTP
PMA + α-CT1 RIS
PMA + α-CT1
shPKCμ B
0 hr
16 hr
B
**
1.0
0.8
0.6
0.4
0.2
0.0
Wound Area (%)
PMA:
ANTP:
α-CT1 RIS:
α-CT1:

Figure S7: Inhibition of Cx43 with α-CT1 increases wound healing in shPKCμ treated cells, related to Figure 7. (A) Representative images of *in vitro* wound healing assay at 0 and 16 hours on HaCaT cells transduced with an shRNA directed against PKCμ treated with PMA (10 nM) in the absence or presence of ANTP, α-CT1 RIS, or α-CT1 peptides (each at 100 μM). Scale bar = 100 μm. (B) Percentage of wound area remaining after 16 hours from three replicate experiments as shown in (A). All calculations are based on three replicates -/+ S.D. **p<0.01 (Student's t-test).
